# SMARTbot: A Behavioral Analysis Framework Augmented with Machine Learning to Identify Mobile Botnet Applications

**DOI:** 10.1371/journal.pone.0150077

**Published:** 2016-03-15

**Authors:** Ahmad Karim, Rosli Salleh, Muhammad Khurram Khan

**Affiliations:** 1 Department of Computer Systems and Technology, University of Malaya, Kuala Lumpur, Malaysia; 2 Center of Excellence in Information Assurance (CoEIA), King Saud University, Riyadh, Kingdom of Saudi Arabia; University of South Australia, AUSTRALIA

## Abstract

Botnet phenomenon in smartphones is evolving with the proliferation in mobile phone technologies after leaving imperative impact on personal computers. It refers to the network of computers, laptops, mobile devices or tablets which is remotely controlled by the cybercriminals to initiate various distributed coordinated attacks including spam emails, ad-click fraud, Bitcoin mining, Distributed Denial of Service (DDoS), disseminating other malwares and much more. Likewise traditional PC based botnet, Mobile botnets have the same operational impact except the target audience is particular to smartphone users. Therefore, it is import to uncover this security issue prior to its widespread adaptation. We propose SMARTbot, a novel dynamic analysis framework augmented with machine learning techniques to automatically detect botnet binaries from malicious corpus. SMARTbot is a component based off-device behavioral analysis framework which can generate mobile botnet learning model by inducing Artificial Neural Networks’ back-propagation method. Moreover, this framework can detect mobile botnet binaries with remarkable accuracy even in case of obfuscated program code. The results conclude that, a classifier model based on simple logistic regression outperform other machine learning classifier for botnet apps’ detection, i.e 99.49% accuracy is achieved. Further, from manual inspection of botnet dataset we have extracted interesting trends in those applications. As an outcome of this research, a mobile botnet dataset is devised which will become the benchmark for future studies.

## Introduction

**Fig 1:**
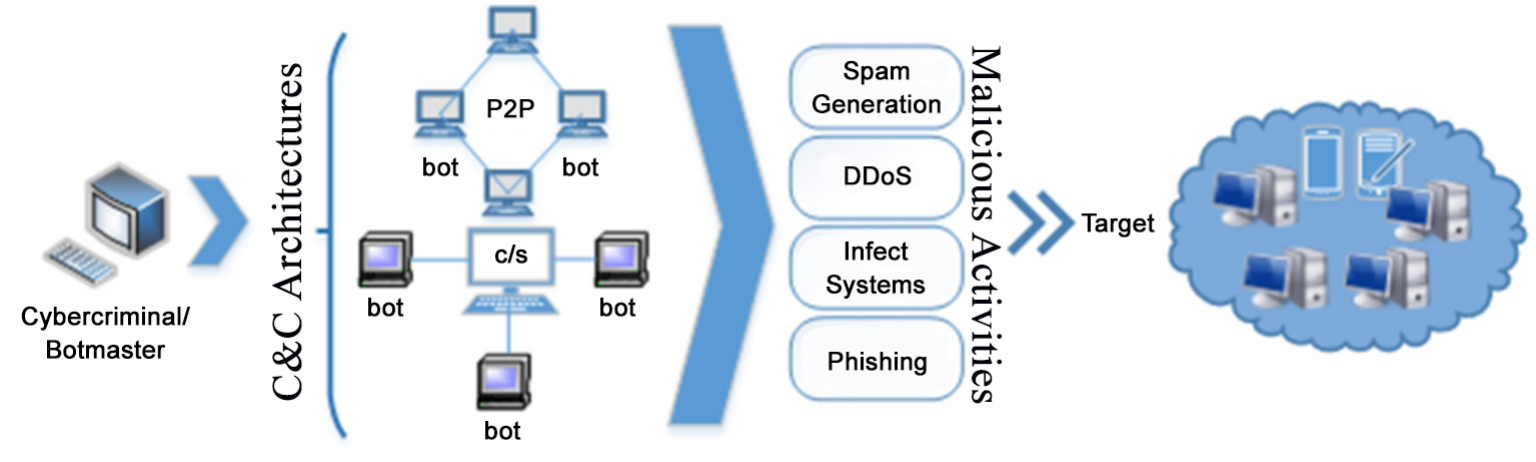
Basic Botnet Architecture.

Botnet refers to a coordinated activity possibly with some malevolent intension in order to perform certain tasks. The working architecture of a mobile botnet is shown in Fig 1. The entities associated with a botnet attack include: bots and Command and Control (C&C). Bots in case of mobile botnet are smartphones, tablets or handheld devices which belong to a particular botnet and are infected by a self-replicating backdoor program. Eventually, it enables a pathway for cybercriminals to control devices remotely and execute commands to perform illegitimate actions. Meanwhile, cybercriminals use a platform i.e. C&C in order to control/instruct bot enemies, execute commands, disseminate malware code and expand bot network. Precisely, this illustration of mobile botnet suggests that the ultimate goals of a mobile botnet vendor are similar to previous generation of PC based botnet i.e. to manipulate personal information of a user, steal financial account particulars, acquire root privileges, generate massive spam and phishing attacks to user’s contact addresses, launch Distributed Denial of Service (DDoS) attacks to turn down the legitimate websites, start enormous hidden processes to perform ad-click fraud without user knowledge and to mine crypto-currencies. The only difference between mobile botnet and PC based botnet is the operational environment/platform within which it executes.

In the past few years, several mobile botnets, such as NotCompatible.C, Zues botnet, DroidDream, BMaster, and TigerBot, have evolved to hinder the performance of smartphone devices. The Zues botnet also affects the Symbian platform. A recent report [1] stated that a variant of the existing malware NotCompatible called NotCompatible.C, which has remote administration capabilities, targets Android devices. The report mentioned that NotCompatible.C is the most dangerous mobile malware with traditional PC-based botnet capabilities ever introduced. Compared with other sophisticated botnets (e.g., Obad, DroidDream, and Geinimi), NotCompatible.C discriminates itself by having a P2P C&C architecture and by employing numerous evasion techniques. Moreover, it offers cross-platform compatibility by sharing its C&C system with Windows bots. Other advancements in botnets include Zeus botnet [2], which affects Android, Symbian, Blackberry, and Windows users, unlike DroidDream botnet [3], which is particularly designed only for Android devices. IKee.B [4] botnet, which scans the IP addresses of target victims, is designed for iPhones, whereas BMaster [5] and TigerBot [6] particularly aim to disrupt Android-based devices. According to [7], Obad botnet has the most sophisticated design as it can exploit several unexplored vulnerabilities in Android OS. Its C&C communication channel is implemented through SMS and HTTP protocols. Moreover, Obad propagates its attack through fake Google Play stores and untrustworthy third-party Android app stores. Given the race among mobile botnet authors, various off-the-shelf mobile malware tools [8] that can perform specific malevolent actions on the behalf of attackers have been introduced. A report published by Forbes [9] states that 97% of mobile malware has an Android architecture. Therefore, botnets are expected to perpetuate their severe effects on the mobile domain in the future.

The two common types of mobile malware analysis approaches include static or code-based and dynamic or runtime execution analyses. Static or code-based analysis does not require the execution of a malware program code; in this analysis, related features are extracted either by directly fetching from the executables [10,11] or by disassembling the program code [12,13]. In addition, high-level structural properties, such as CFGs or FCGs, are also extracted from the disassembled code of the malware binaries and utilized as the primary source of information for malware detection [14,15]. By contrast, dynamic analysis-based systems require malware binaries to be run in a virtual environment called sandbox to monitor the execution traces of these malware binaries and fetch their runtime behavior, such as API calls or system calls, for further analysis and detection [16–18]. As described earlier that mobile botnet is somewhat different from mobile malware because of the involvement of the C&C system in the former. However, the majority of existing detection solutions target mobile malware in general. Thus, we proposed in [19] a static analysis approach to detect mobile botnet applications.

SMARTbot is a dynamic analysis framework composed of three basic components: dynamic analysis, feature mining, and learning. Initially, Android applications are inputted into the dynamic analysis component using the prebuilt cloud-based malware analysis platform called Andrubis [20]. The outcomes of the dynamic analysis component are trace and log files in XML and PCAP formats; these files are transmitted to the feature mining component. In the feature mining process, various behavioral properties and features, which are particular to a botnet attack, are observed and extracted. Finally, in the learning component, an ANN backpropagation model is applied to the unlabeled Drebin dataset to train the botnet detection classifier. Subsequently, six machine-learning classifiers (i.e., BayesNet, SVM, multilayer perceptron (MLP), simple logistic regression, J48, and Random Forest) are applied and evaluated using the labeled Drebin dataset [21] to select the optimum classifier model.

We are unaware of a system that can identify botnet features in suspicious mobile applications through the dynamic observation of infected binaries. Similarly, to date, no study has been conducted to distinguish botnet features in existing mobile malware binaries through dynamic analysis augmented by machine-learning techniques. Therefore, this study explicitly leverages the identification of Android-based mobile botnet applications using dynamic analysis integrated with machine-learning algorithms. The current study’s contributions highlighted in this paper are as follows:

1. We propose SMARTbot, a robust systematic framework based on the dynamic analysis of Android applications augmented with machine-learning techniques, to distinguish botnet behavior in malicious mobile applications.
2. This study identifies the critical features of malicious mobile applications; these features enable these applications to initiate and persist and eventually conduct a mobile botnet attack. Specifically, C&C communication patterns in malicious mobile applications are investigated through behavioral signatures.
3. The most challenging task in any machine-learning system is to correctly label class attributes. We performed class labeling (botnet or malware) by using ANN’s backpropagation algorithm.
4. The proposed classifier model was evaluated with six existing machine-learning classification algorithms (i.e., BayesNet, SVM, MLP, simple logistic regression, J48, and Random Forest). Simple logistic regression was selected as the best classification algorithm that can effectively identify botnet applications from the malicious corpus.
5. Various interesting properties pertaining to an HTTP-based mobile botnet attack were manually investigated.
6. To assist the research community, we uploaded the necessary codes, classifier models, and generated mobile botnet dataset to a public repository [22].

Consequently, we determined that applications that belong to a specific botnet family demonstrate certain C&C communication patterns. Specifically, each malware application belonging to a particular family performs similar actions while executing remote commands[23,24], sharing information, and implementing request/response mechanisms. Thus, the main aim of this study is to identify the properties and features that can lead to a botnet attack by conducting behavioral observations, distinguishing botnet from other malicious applications, and employing machine-learning approaches.

The rest of the paper is organized as follows: Section-2 discusses the related work, Section 3 describes the working architecture of our proposed detection approach, Section 4 presents cross validation results. Section 5 and 6 presents the comparative analysis of botnet and malware applications and performance evaluation respectively. Section 7 summarizes and concludes this paper.

## Related Work

A dynamic taint tracking system TaintDroid was proposed by [25] to track vulnerabilities in Android systems with four granularities of taint disseminations: methods, message, variable and file level. The taint tracking system marks sensitive data generated from multiple sources. The main goal of their approach is to mark infected data before leaving the taint sync. However, the approach is limited in scope because it does not track implicit control flows due to performance overhead. Many dynamic analysis approaches also use static analysis as a prerequisite to recognize potentially harmful actions. Static analysis is helpful in minimizing resource overhead during large scale dynamic analysis. Based on this analogy, SmartDroid [26] was designed to automatically identify UI-specific conditions which cause malicious activities. Initially, it generates Activities and Function Call Graphs for each application by using static analysis. Later each application is passed through a dynamic analysis procedure to obtain a sequence of UI-specific events causing the execution of sensitive API. As the system only triggers UI events, thus, activities generated as background process cannot be traced. Likewise, another study [27] states that many malicious activities are initiated by the services running at backend.

In a hybrid analysis approach DroidRanger [28], the applications are first scrutinized based on their dangerous permission usage. Next, the behavior of these applications is compared with known malware samples on the basis of applications’ manifest, used packages, function call graphs and code architecture. In addition to that, applications with untrusted code are treated as zero-days and are further analyzed by the system. However, the system does not cover tracking of network communication of these applications. Another dynamic analysis scheme VetDroid [29] is designed to examine the permission usage of each application and the analyzed applications are executed in a secure sandbox for a certain time. This approach uses Monkey runner [30] to trigger UI events. A permission analysis component of VetDroid extracts all permissions and highlights the connections between them. As a result, the system generates a function call graph through which malicious applications are identified. DroidBox is a sandbox for behavioral analysis, proposed by Lantz [31], which can effectively analyze Android applications. However, it lacks in executing applications prior to Android version 4.2. Both DroidBox and TaintDroid are available as open source packages.

Few approaches [32,33] employ standard machine learning classifiers to detect Android malware whereas, a tool DroidAPIMiner was proposed by [33] to detect malicious application by measuring the frequency of APIs called by each application. They conclude that the rate with which APIs are called in applications with conditions on parameters is 6% higher in malware than benign applications. Further, the authors applied standard machine learning classifier KNN to verify their claim.

Another approach, MAST [34], which uses Multiple Correspondence Analysis (MCA) to compute the distance between analyzed application and predefined group of malicious applications. The features extracted for comparison include: permissions, intent filters, zip archives, and native code. The approach results in a general rank showing the possibility of malicious actions in mobile applications. The system is specifically designed for analysis of large scale market stores. A machine learning based hybrid detection and classification approach is proposed by[35]. The authors built their software using an open-source framework known as CuckooDroid. The approach is comprised of anomaly detection engine and misuse detection engine by static and dynamic analysis approaches. The system is evaluated on Drebin dataset, however the system is still underway for cloud based production environments.

A more recent study MARVIN [36] is proposed to investigate malicious Android applications with the help of machine learning classifiers which assists user to predict maliciousness in applications by generating a malice score for each observed application. It is an off-devise analysis system and its results strongly rely on the output of already developed cloud based sandbox service known as Andrubis. This work is somewhat related to our approach. However, we aim to identify bot application binaries, whereas the said approach is used for mobile malware detection in general. Moreover, we used Andrubis only for data acquisition. Furthermore, MARVIN is a generic toolkit to identify maliciousness in Android application with the help of SVM, L1 and L2-regulerzation classification algorithms, whereas our approach is particular to identification of C&C enabled Android applications with the help of ANN’s back-propagation modeling.

## Proposed Framework

**Fig 2:**
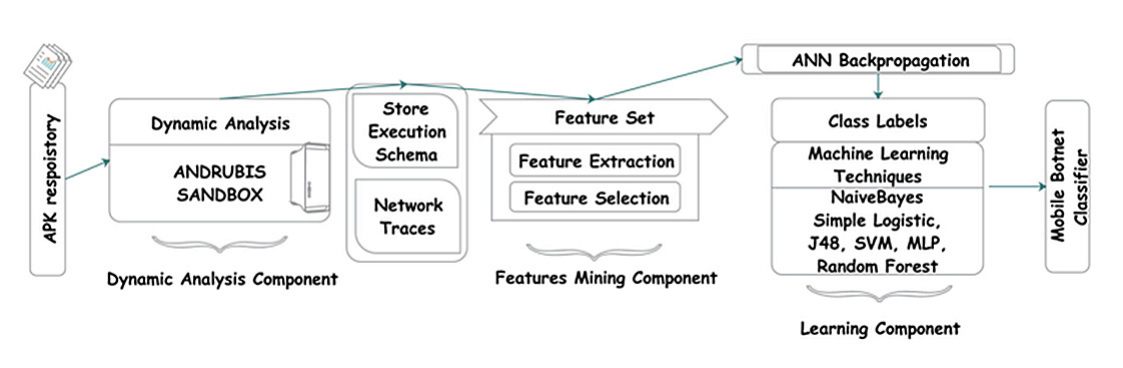
SMARTbot Framework Overview.

We propose SMARTbot, a framework which learns to distinguish applications having C&C functionality from malicious corpus through dynamic analysis of Android applications. Our framework is purely based on machine learning techniques that can classify applications based on various features collected at runtime. Among the various features, the more prominent ones we selected are opened connections, reading/writing data using network operations, started services, cryptographic operations, HTTP traffic sent/received, DNS requests, and SMS sent/received. Fig 2 shows the basic architecture of the SMARTbot framework together with the component hierarchy.

The proposed system is based upon passive analysis. Therefore for data collection phase, we consider the following sources: (a) 3rd party market store, (b) google market store, (c) and Drebin dataset. The framework consists of three major components: Dynamic Analysis Component, Features Mining Component, and Learning Component. This study leverages our previous study [19] in order to obtain concrete observations with the help of dynamic analysis and machine learning techniques to detect malicious mobile applications having C&C features with more accuracy.

### Dynamic Analysis Component

In SMARTbot’s Dynamic Analysis Component each application is executed in a sandbox in order to observe applications’ behavior at runtime. Currently, various off-the-shelf dynamic analysis tools are available with desktop or cloud based[37,38] environment. For example, AndrubisAndrubis [20], DroidBox [31], DroidScope [39], APK Analyzer [40], or APKScan [41] are some of the tools recently adopted and tested by research and development community. DroidBox is an open source package for dynamic analysis which cannot be used explicitly for large datasets because of its limited resultant parameters and deficiency to execute latest Android applications. In contrast, Andrubis provides an automated cloud based malware analysis platform which can generate reports with rich parameters (static and dynamic). Therefore, we have selected Andrubis (SaaS) sandbox in order to execute and collect network traces. Through an automated script we have uploaded the whole dataset of Drebin to Andrubis and obtained the dynamic analysis results in XML files. After downloading each application report, SMARTbot has further analyzed those reports in later stages and labeled them accordingly. As an additional step to dynamic analysis, collection of network traces are also required to identify any potential remote access feature which is discussed in Section (Future work).

### Feature Mining Component

Feature selection and feature extraction plays a vital role in learning based systems. Both of these tasks are performed in our Feature Mining Component. As the system only deals with dynamic code analysis, therefore we are only considering properties pertaining to run-time analysis of applications. For a better learning system, selected features reflect the truthfulness of that system. For this purpose, we studied the behavior of various known botnet applications/families in a nutshell followed by execution of various samples from previously identified botnet families onto sandbox and extraction of dynamic features associated with them. Subsequently, our system learns this behavior pertaining to botnet applications and compares it with other kinds of malware attacks (e.g, spam, banking Trojan, premium-SMS, and device outage attack etc). As a result, we have classified our datasets into two domains: one of them is labeled botnet dataset and the other is unlabeled Drebin dataset which will be labeled using ANN’s backpropagation model in Learning Component. These labels will eventually be used for evaluation (i.e testing).

#### Dataset Used

SMARbot uses different datasets for training and testing. The datasets chosen for analysis and evaluation purpose is presented in Table 1.

**Table 1:** Dataset Used.

Dataset	Total Samples	Total Botnet/%	Total Malware/%	# of Features
Sample Botnet	36	23/62%	14/38%	16
Drebin	4891	3145/64%	1746/36%	16

In order to learn runtime behavior of botnet applications we have chosen 36 malicious applications that belong to 49 different malware families [21].

Among the selected applications, 62% represent known botnets, whereas the remaining 38% belong to other malware families without the enabled C&C feature. A short summary of the selected sample dataset is presented in Table 2. We labeled this sample dataset (either malware or botnet), which became the baseline for the dynamic feature selection and was used to train our neural network model. Ultimately, our framework employs the same sample set for learning the behavioral properties of botnet applications. After executing these applications in a sandbox, we collected the features that are most relevant to a botnet activity. The execution time for feature selection was 2 minutes, and the resultant schema was stored in a CSV file for further analysis using a Python script. For the evaluation, we selected the Drebin dataset because it is currently the largest malware dataset that is publicly available. This dataset is unlabeled and is up for labeling based on our class-labeling criteria discussed in Section 3.

**Table 2:** Summary of Selected Sample Dataset.

No	Malware Family/Name	Operational Impact	Year Introduced	C&C	Category
**1**	NotCompatible.C [1]	a) Uses a two-tiered C&C architecture b) The gateway C&C server works as a load balancer, which has the responsibility to filter and segment geographically disperse IP address regions of infected devices and allows legitimate clients to connect.c) This kind of architecture is hard to discover by existing dynamic analysis approaches.	2015	DNS	Mobile Botnet
**2**	FakeNotify [42]	a) sends SMS messages to premium numbers. b) collects and sends user information. c) download applications	2012	SMS	Premiums MS
**3**	HijackRAT [43]	privacy leakage, theft of banking credential	2014	SMS	Banking Trojan
**4**	Hippo [44]	a) Sends SMS messages to a premium rated number. b) deletes the incoming SMS messages	2014	SMS	Premium SMS
**5**	Opfake [45]	send premium SMS messages	2012	SMS	Premium SMS
**6**	Obad [7]	a) sends SMS messages to premium numbers b) download other malicious programs and install on user device without user notice c) Spread these infected programs using Bluetooth.	2013	HTTP	Mobile Botnet
**7**	DroidDream [3]	a) It uses two known exploits, *exploid* and *rageagainstthecage*. b) Instructed by C&C	2011	HTTP	Mobile Botnet
**8**	Geinimi [46]	Once the malware is installed on a user's phone, it has the potential to receive commands from a remote server that allows the owner of that server to control the phone.	2011	HTTP	Mobile Botnet
**9**	Plankton [47]	Works similar to as IRC Spam Bot	2011	HTTP	Mobile Botnet
**10**	SpamSoldier [48]	The infected device connects to C&C and receive instructions: a) The SMS spam message and; b) A list of 100 US phone numbers to spam;	2012	SMS/HTTP	Mobile Botnet
**11**	DroidKungFu [49]	a) Extract user’s device information (IMEI, OS version, device version etc.) from infected device. b) Store this information on a separate file and sends this file to remote host for further instruction.	2011	HTTP	Mobile Botnet

#### Feature Selection

SMARTbot uses the dynamic feature space and selects the features which show the behavior of mobile applications in terms of botnet actions, as presented in Table 3. As a result, we selected the features with respect to file system activities, network connections, information leakage, started services, SMS, cryptographic operations, DNS request, HTTP traffic parameters and unknown TCP and UDP conversations, majority of these properties are already proven as the causes of traditional PC and mobile botnet attacks [27,50–57].

**Table 3:** Feature Vector.

Features	Parameters	Rationale
**File Activity**	Read/Write	Write/Read file system to and from SD card.
**Network Operations**	a) Opened Connections b) Network Read c) Network Write	Establish and persist remote connection by the malicious application.
**Information Leaks**	a) Network Leaks b) File Leaks	This parameter observes the network and file leaks on the network.
**Services**	Started Services	Background services started by malicious applications.
**SMS**	a). Sent SMS b) Received SMS	To identify SMS based botnets this feature is very import to consider.
**Cryptography**	Cryptographic Operations	Crypto operations are performed by malicious writers in order to minimize the code coverage.
**DNS Traffic**	DNS Requests	Frequency DNS requests indicates a botnet attack.
**HTTP Traffic**	a) HTTP Conversations b) HTTP Connection Attempts	HTTP based botnet uses this features to establish TCP based connection with outside word.
**Unknown Conversation**	a)TCP Conversation b) UDP Conversation	Establishment of connection with use defined TCP or UDP ports should also be noted.

#### Feature Extraction

As part of dynamic analysis component, we need to extract only those features which are most appropriate for an application to initiate a botnet attack. For this purpose, we bind a program with the API provided by [20], execute each malicious binary in an automated fashion on publically available cloud based sandbox and collect run-time execution traces of each application. This service executes program instructions through a modified Dalvik VM deployed virtual machine introspection (VMI) for system-level inspection. In addition to that, a rich external stimulation is implemented to capture maximum program behavior and to increase code coverage [30]. Average running time of each application is 3 to 5 minutes depending upon the instruction set. After collecting all reports which are stored in XML file format, we need to extract features mentioned in Section 3. For this purpose, we devise a python program logic which can automatically extract botnet features and stores in a CSV file for further analysis. For this purpose, we have used element tree xml API of python [58,59] and regular expressions to build the said feature vector.

During the specified running time we have collected the frequencies of feature vector called by those applications. For instance, how many total DNS requests are initiated by an application? Similarly, what is the total number of opened HTTP connections in order to establish C&C communication? etc.

### Learning Component

Machine learning and data mining are extensively used in anomaly detection especially in establishing generic and heuristic methods [60]. Data mining is on top of the machine learning to device methods for prediction, classification, inference and regression. Ultimately, selection of an appropriate method depends on the nature of application. In our study, we are selecting classifier based on feature length, performance, number of classes and ranking criteria.

We have chosen simple logistic regression, NaiveBayes, RandomForest, SVM, MLP and J48 as our classification algorithms to build and test the generated classification model. A short description of these algorithms is presented in the next subsection. Training set consists of malicious samples not having C&C properties and well-known mobile botnet applications. As the system is specific for botnet detection, therefore we have selected features which are most relevant to a botnet life cycle which includes connection, infection and resilience. Consequently, training function computes the conditional and marginal probabilities in order to formulate algorithm for the final classification decision.

### Class Label Assignment Criteria

Initially, we have learned against the known botnet and malware applications to identify their behavioral patterns with the help of features discussed in feature mining component. On the same grounds we need to train Drebin dataset in order to correctly classify botnet application from malicious corpus. For this purpose, we have adopted ANN’s backpropagation model to assign class labels to Drebin dataset and attained high accuracy. The reason to choose ANN’s backpropagation modeling is that, during our initial classification results, MLP outperform in terms of accuracy, precision and recall rates when applies to sample dataset. Similarly, MLP utilizes backpropagation as a supervised learning technique to train the network. Therefore, we have opted the ANN’s backpropagation modeling to classify Drebin dataset.

**Fig 3:**
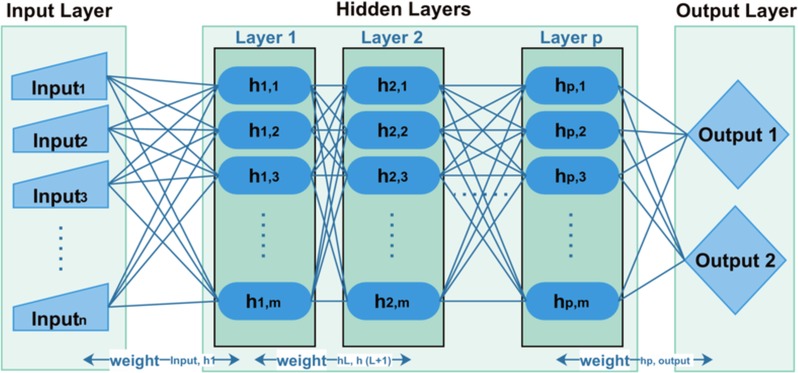
Backpropagation model.

Backpropagation is a commonly used neural network for supervised training of multilayered neural networks [61]. During execution, a non-linear relationship is created between input and output patterns so that more accurate output from incoming training patterns is achieved. For this purpose, backpropagation adjusts the internal weights and revises all weights among each layer. Backpropagation is a learning algorithm where operations are taken place in two steps: Forward Pass and Backward Pass, as shown in Fig 3.

In our case, the number of inputs in input layer is the total number of features we have nominated that is 16. Similarly, the output layer contains two predicted outputs i.e malware and botnet.

Backpropagation performs operations in two different steps: forward pass or feedforward and backward pass or backpropagation. Forward Pass step receives input pattern via nodes at input layer and keeps forwarding input pattern from each of the multi-layer hidden nodes until it reaches nodes at output layer where output is generated. During Forward Pass, pre-defined weights are used to process input pattern throughout the network from input layer towards output layer. Once the output is obtained from nodes of output layer, each output node generates error signal by comparing obtained output with expected output. This error signal will further be utilized by second step of Backpropagation

1. For first hidden layer
  $${Net\_Input}_{h_{1.j}}=\left[\sum_{i=1}^{n}{weight}_{{input}_{i}↔h_{1,j}}\times {input}_{i}\right]$$
  $$Squash\left({Net\_Input}_{h_{1.j}}\right)=\frac{1}{1+e^{-({Net\_Input}_{h_{1.j}})}}$$
2. For hidden nodes of each next hidden layer *L*
  $${Net\_Input}_{h_{L.j}}=\left[\sum_{i=1}^{n}{weight}_{{input}_{i}↔h_{L,j}}\times Squash({Net\_Input}_{h_{L-1.j}})\right]$$
  $$Squash\left({Net\_Input}_{h_{L.j}}\right)=\frac{1}{1+e^{-({Net\_Input}_{h_{L.j}})}}$$
  where: *j* = 1,2,3,…,*m* for corresponding layer
3. For each output node *o*
  $${Net\_Output}_{o}=\left[\sum_{j=1}^{P,m}{weight}_{h_{P,j}↔{output}_{o}}\times Squash({Net\_Input}_{h_{P.j}})\right]$$
  $${Obtained\_Output}_{o}=\frac{1}{1+e^{-({Net\_Output}_{o})}}$$
  Where:
  P is last hidden layer

Finally, Backward Pass is performed to update weights throughout the network. Backward Pass is initialized at output layer and carried out by propagating error signals backwards from output layer to each hidden layer until input layer. As all hidden nodes have collectively contributed in obtained output, they all have effect on generated error signals. Error signal is now propagated to each node of immediate hidden layer and new weights for the links connecting this hidden layer to output layer are calculated. In the same way weights between each layer are calculated relative to their contribution in error signals. These updated weights are assumed to show minimum error for later training patterns. Thus, the aim of Backpropagation to solve learning problem is achieved.

1. For weights between last hidden layer and output layer
  $${Optimized\_weight}_{{output}_{o}{↔h}_{P,j}}={weight}_{{output}_{o}{↔h}_{P,j}}-\eta \times \frac{\partial (Error)}{\partial ({weight}_{{output}_{o}{↔h}_{P,j}})}$$
  where *η* is learning rate,
  $$\frac{\partial (Error)}{\partial ({weight}_{{output}_{o}{↔h}_{P,j}})}=\frac{\partial (Error)}{\partial ({Obtained\_Output}_{o})}\times \frac{\partial ({Obtained\_Output}_{o})}{\partial ({Net\_Output}_{o})}\times \frac{\partial ({Net\_Output}_{o})}{\partial ({weight}_{{output}_{o}{↔h}_{P,j}})}$$
  And
  $$Error=\sum_{o=1}^{2}\frac{1}{2}{\left({Expected\_Output}_{o}-{Obtained\_Output}_{o}\right)}^{2}$$
2. For weights between hidden layers
  $${Optimized\_weight}_{h_{L,j}{↔h}_{L-1,j}}={weight}_{h_{L,j}{↔h}_{L-1,j}}-\eta \times \frac{\partial (Error)}{\partial ({weight}_{h_{L,j}{↔h}_{L-1,j}})}$$
  where:
  $$\frac{\partial (Error)}{\partial ({weight}_{h_{L,j}{↔h}_{L-1,j}})}=\frac{\partial (Error)}{\partial (Squash(h_{L,j}))}\times \frac{\partial (Squash(h_{L,j}))}{\partial ({Net\_Input}_{h_{L.j}})}\times \frac{\partial ({Net\_Input}_{h_{L.j}})}{\partial ({weight}_{h_{L,j}{↔h}_{L-1,j}})}\;\text{and}$$
  $$\frac{\partial (Error)}{\partial (Squash(h_{L,j}))}=\sum_{o=1}^{2}\frac{\partial (Error)}{\partial ({Obtained\_Output}_{o})}\times \frac{\partial ({Obtained\_Output}_{o})}{\partial ({Net\_Output}_{o})}\times {weight}_{{output}_{o}{↔h}_{P,j}}$$
3. For weights between first hidden layer and input layer are:
  $${Optimized\_weight}_{h_{1,j}{↔input}_{i}}={weight}_{h_{1,j}{↔input}_{i}}-\eta \times \frac{\partial (Error)}{\partial ({weight}_{h_{L,j}{↔input}_{i}})}$$

## Cross Validation

In this section, we will present the classifier validation results that are collected by applying machine learning classifiers to labeled Drebin dataset.

### Classifier Evaluation

As discussed before, our main objective of this system is to build a classification model which classifies malicious applications having C&C features with other types of malwares. For this purpose, we have used six different types of machine learning classifiers: SVM, Naïve Bayes, Random Forests, J48, MLP and simple logistic regression. These classifiers belong to different classifications families. J48 [62] belongs to the C4.5 Decision Trees [63] which is used for generating a pruned or unpruned model. Similarly, Random Forest classifier decides the predictor’s accuracy by constructing various random trees. Moreover, simple logistic regression is based on Logistic Model Trees [64], whereas the Naïve Bayes is derived from Bayes Theorem [65] with the predictors having independence assumptions. In SMARTbot framework, the reason to use different classifiers with diverse logic is to produce higher classification accuracy. We observed that, MLP performs well in terms of accuracy, precision, recall and F-measure for Sample dataset, on the other hand, simple logistic regression outperforms in terms of classification accuracy, precision, recall and F-measure for Drebin dataset which we have labeled based on the method described in previous section.

All the experiments are performed in a powerful feature of Weka workbench [66] known as Weka Experimental [67]. It has a GUI explorer built-in for experimenting machine learning algorithms on big datasets, and robust enough to produce a large number of experimental results needed for evaluation and comparison. Normally, the validation in machine learning classifiers is performed in two different ways to assess accurate performance measures for classifiers. One method is called K- fold cross validation [68] and the other is known as random sampling validation [69].

We have used *K* -Folds cross validation as a process to validate our classifier’s model accuracy and compare the results with random sampling in order to measure the efficacy of our model (section 0). In our case, we used *K* as 10 to perform cross validation tests for our classifier model. Although *K* = 10 is commonly used [70], however *K* is not a fix parameter. To evaluate each classifiers’ performance we used the following standard classification as described in Table 4.

**Table 4:** Observed Classification Parameters.

Parameters	Formula (if any)
True Positive Rate (sensitivity, recall, hit-rate)	$\frac{TP}{TP+FN}$
True Negative Rate (Specificity)	$\frac{TN}{TN+FP}$
False Positive Rate	$\frac{FP}{FP+TN}$
False Negative Rate	$\frac{FN}{TP+FN}$
Accuracy	$\frac{TP+TN}{TP+TN+FP+FN}$
Precision	$\frac{TP}{TP+FP}$
F-Measure	$F=\left(2\times |\frac{percision\times recall}{precision+recall}\right)$
Area Under ROC Curve	-

### Classification Results for the Drebin Dataset

In this section, we used Drebin dataset to validate our results and assess the applicability of our model in a large-scale environment. The steps involved in labeling this dataset are discussed in last section.

**Fig 4:**
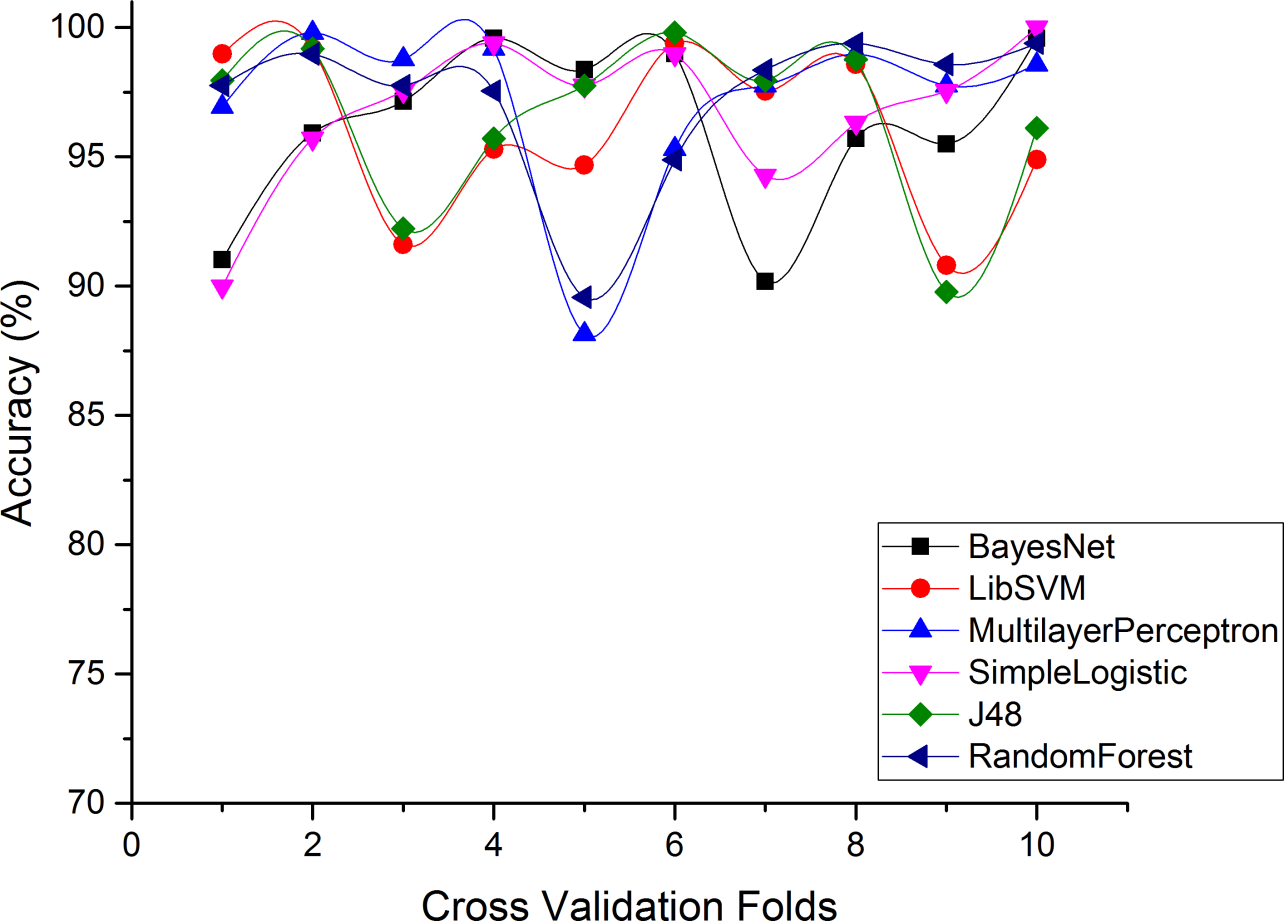
Accuracy of each classifier for Drebin dataset.

**Fig 5:**
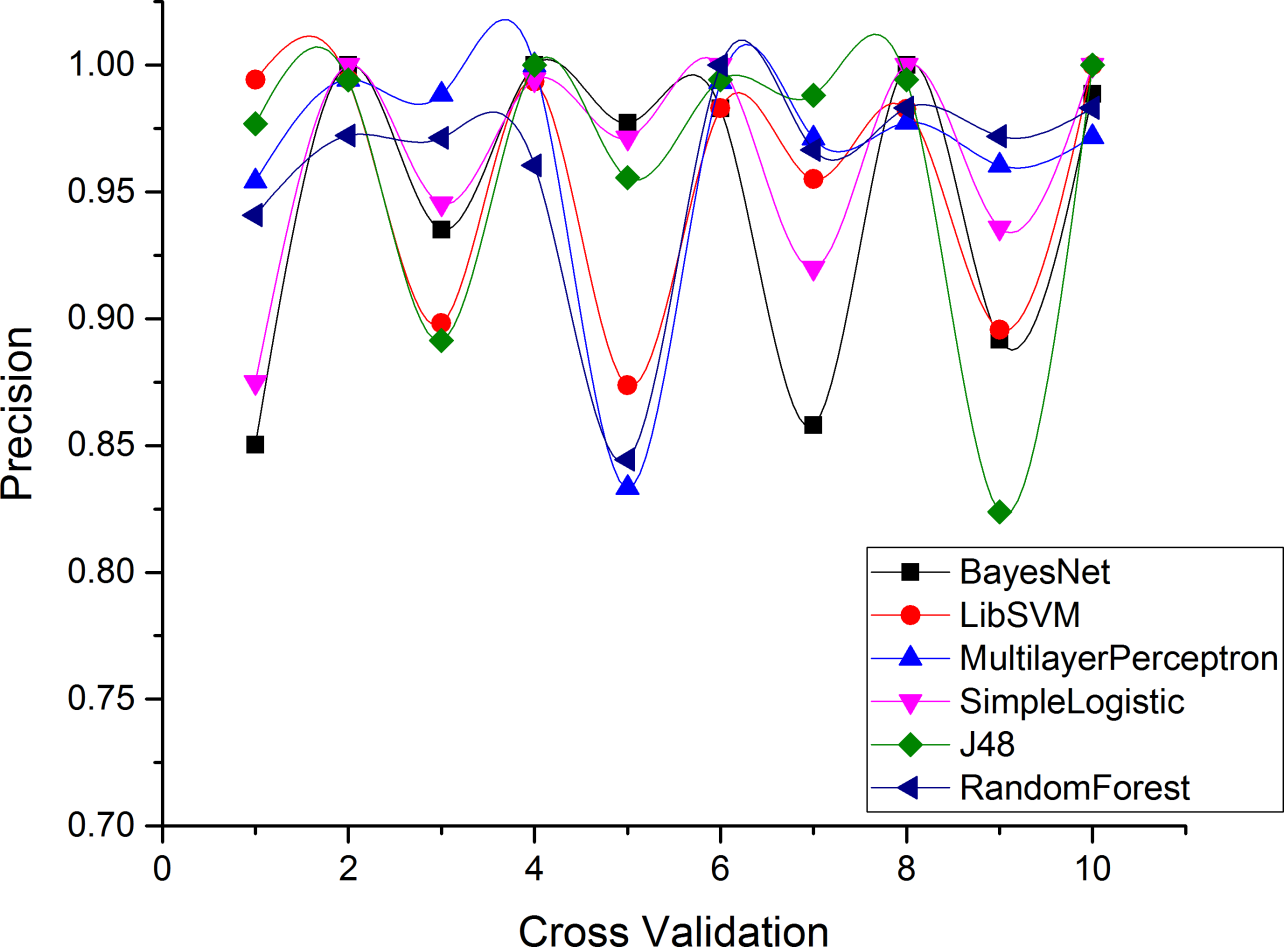
Precision of each classifier for Drebin dataset.

**Fig 6:**
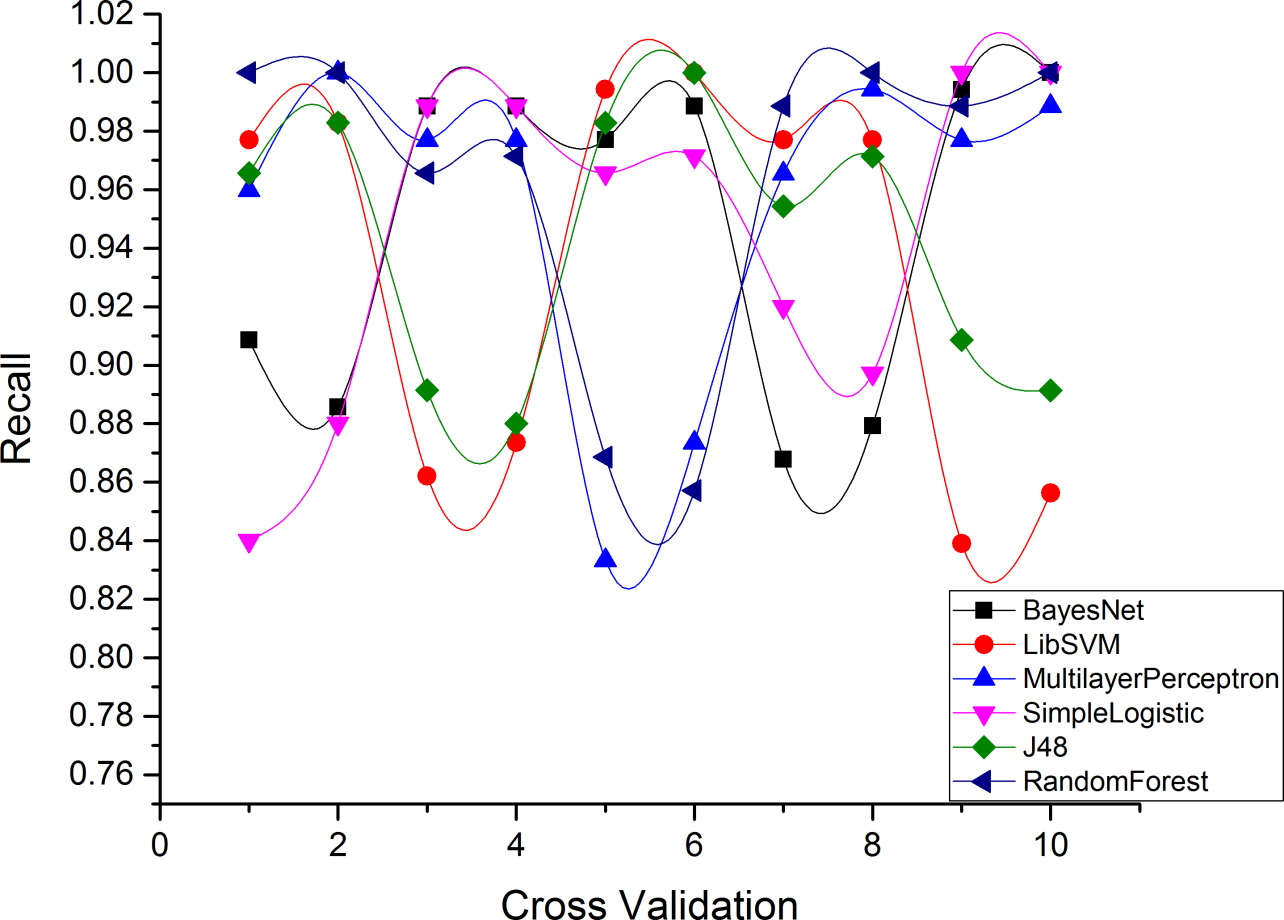
Recall Rate for each classifier in Drebin Dataset.

**Fig 7:**
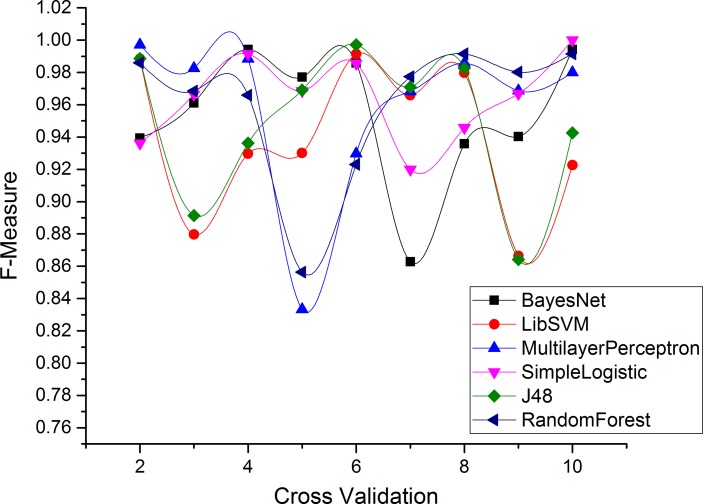
F-Measure for each classifier in Drebin Dataset.

Figs 4–7 and show the accuracy rates (in percent), precision, recall, F-measure for Drebin dataset using 10-fold cross validation. Although all ML classifiers produced relatively good accuracy rates i.e higher than 90% however, simple logistic regression outperforms the other tested classifiers. It correctly classifies 99.49% of Drebin dataset using the selected features to distinguish botnet applications. In difference, Naive Bayes, SVM, MLP, J48 and RF achieve accuracy rate of 91%, 96%, 97%, 98% and 99% respectively. Table 5 also reveals that the precision values support the accuracy rates of the machine learning classifiers in establishing an effective model. The SVM has the maximum precision value than other classifiers. The precision value for the SVM is 1.00 while the precision values for Naive Bayes, MLP, simple logistic regression, J48, and RF are 0.87, 0.94, 0.99, 0.98, and 0.99 respectively. Moreover, results of Recall rate and F-measure for 10-fold cross validation conclude that on the average, the maximum recall rate generated by Simple Logistic which is 1.00, whereas the recall rates for Naive Bayes, SVM, MLP, J48 and RF is 0.87, 0.88, 0.99, 0.97, and 0.98 respectively. Similarly, the highest F-measure of simple logistic regression having 0.99 values other than the obtained results of Naive Bayes, SVM, MLP, J48 and RF. Whereas, the attained F-measure for Naive Bayes, SVM, MLP, J48 and RF are 0.87, 0.93, 0.96, 0.97 and 0.98 respectively.

**Table 5:** Overall Accuracy and Precision for Drebin dataset (rounded).

	Naive Bayes	SVM	MLP	simple logistic regression	J48	RF
**Accuracy (%)**	91	96	97	**99.49**	98	99
**Precision**	0.87	1.00	0.94	**0.99**	0.98	0.99
**Recall**	0.87	0.88	0.99	**1.00**	0.97	0.98
**F-measure**	0.87	0.93	0.96	**0.99**	0.97	0.98

**Fig 8:**
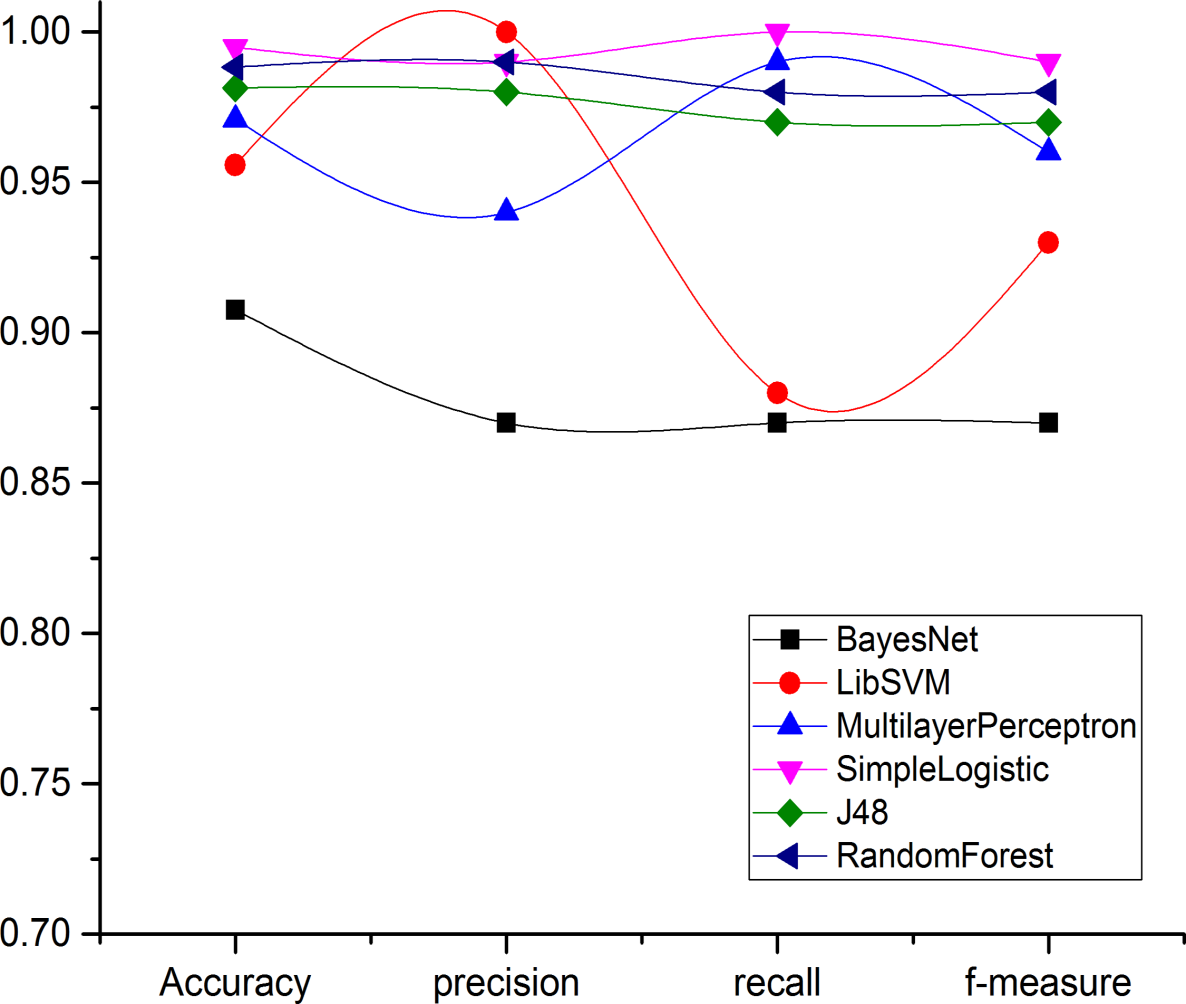
Overall performance comparison against each classifier for Drebin Dataset.

**Fig 9:**
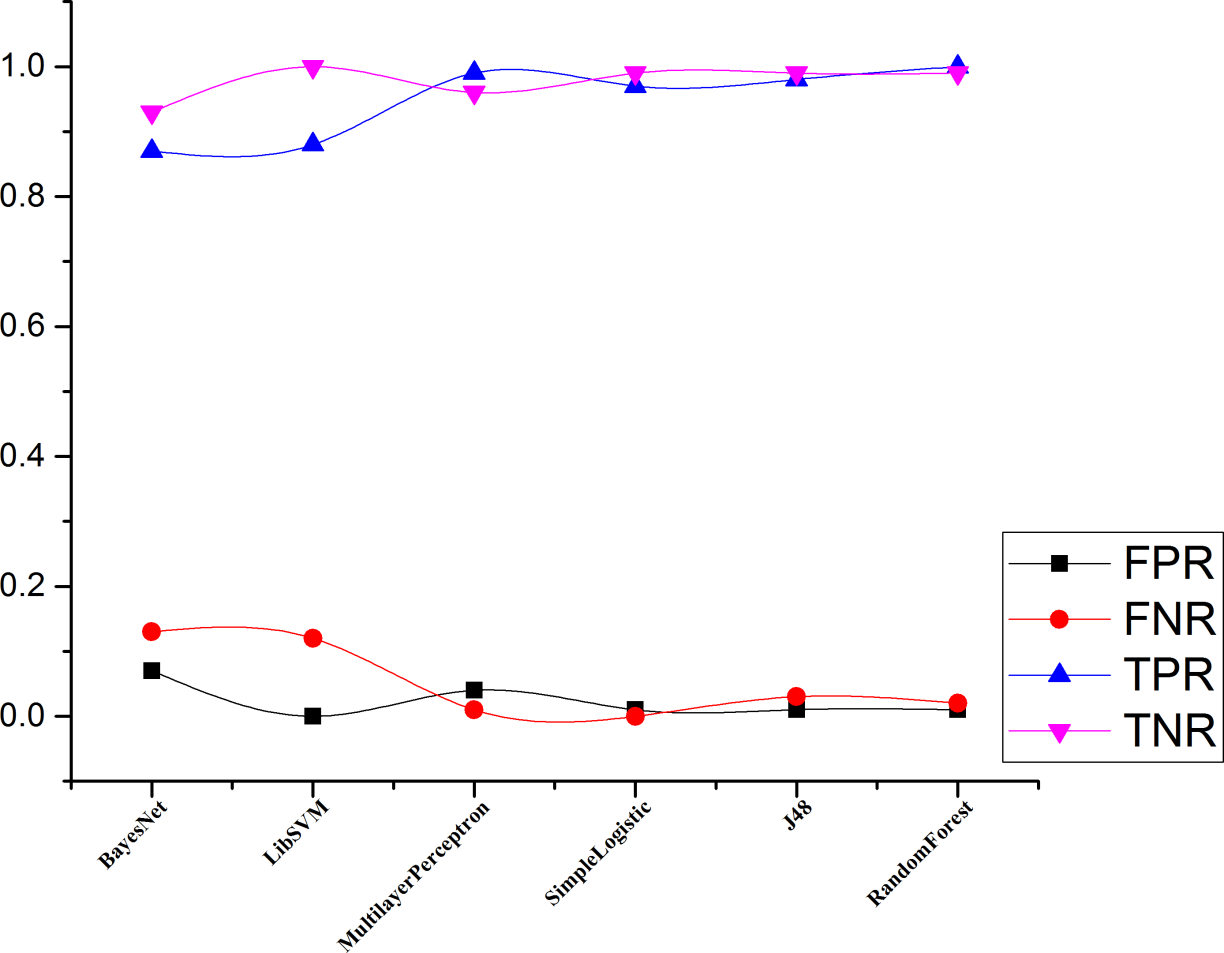
Overall accuracy comparison against each classifier for Drebin Dataset.

Moreover, Figs 8 and 9 derive the overall performance of classification algorithms when applied on Drebin dataset. From the Fig 8, it can be concluded that the simple logistic regression performs the best in terms of accurately classifying the Drebin dataset with 99% using the selected feature vector. Similarly, simple logistic regression has the highest recall rate of 100% from its counterpart classifiers while having the minimum FNR of 0. However, the TPR of MLP is slightly improved than simple logistic regression (0.97) which is 0.99. Moreover, the FNR for Naive Bayes, SVM, J48, and RF are 13%, 12%, 3% and 2% respectively.

## Comparative Features Evaluation

In this section, we will discuss and compare different features with respect to botnet and malware applications in order to evaluate SMARTbot framework.

### Cryptographic Operation Statistics

**Fig 10:**
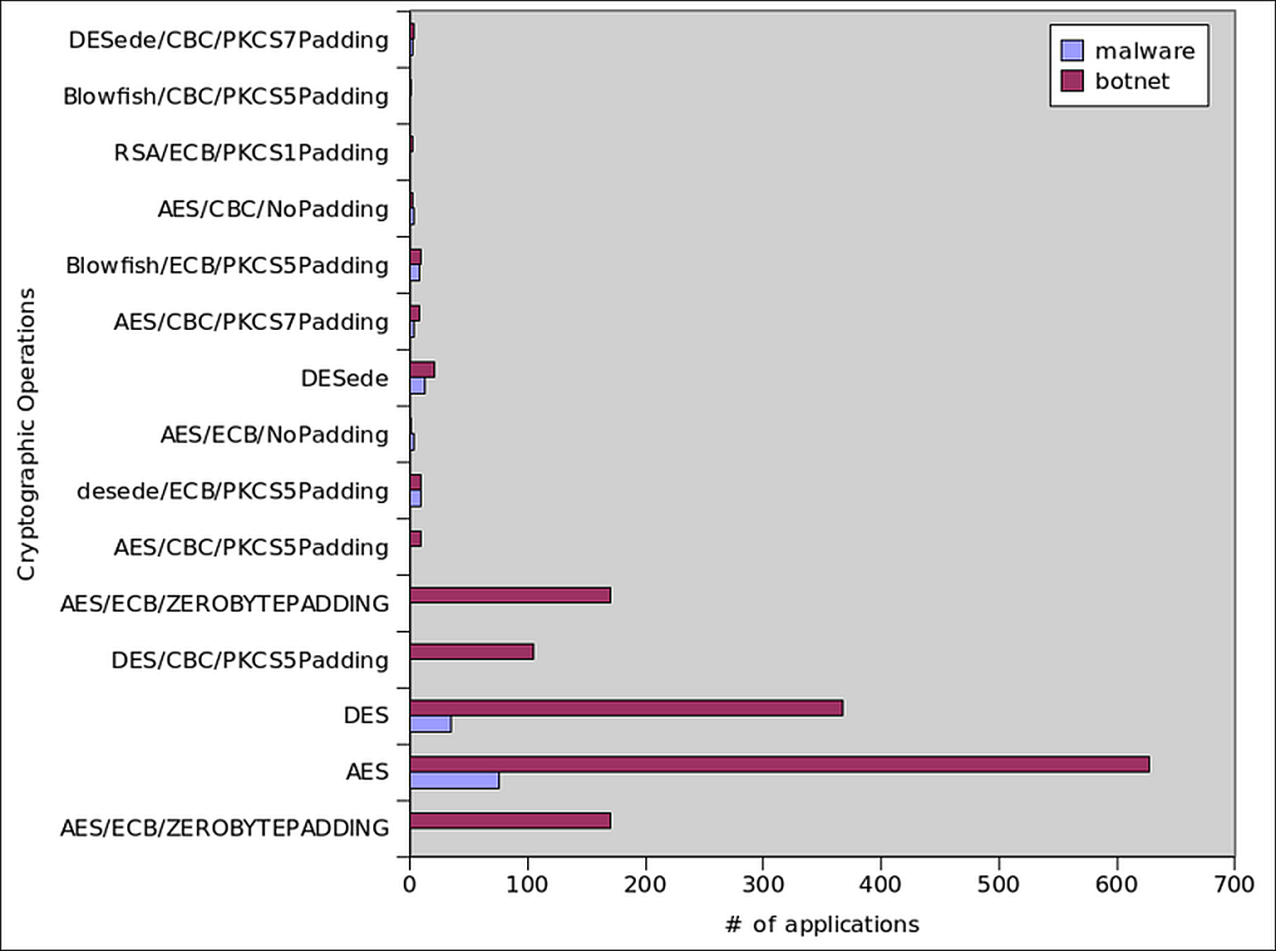
Cryptographic Operations.

Mobile application developers use cryptographic operations which include message authentication codes and block ciphers to secure communication and data. From the Fig 10 we can observe that, the most common cryptographic algorithms observed during the dynamic analysis of botnets were AES (20%), DES (12%), AES/ECB/ZEROBYTEPADDING (5%), and DES/CBC/PKCS5Padding (3%). Malware, on the other hand, mainly used AES (4.3%) and DES (2.0%). According to [71], DES was the predominantly used cryptographic algorithm in 2010 (98%); however, its usage reduced to 1.53% in 2013. After which, malware writers changed their motivation to stronger algorithms like, AES and Blowfish. In our analysis we observed that the Blowfish trend in the botnet applications was only 0.2% in all samples and no malware sample used this algorithm.

**Fig 11:**
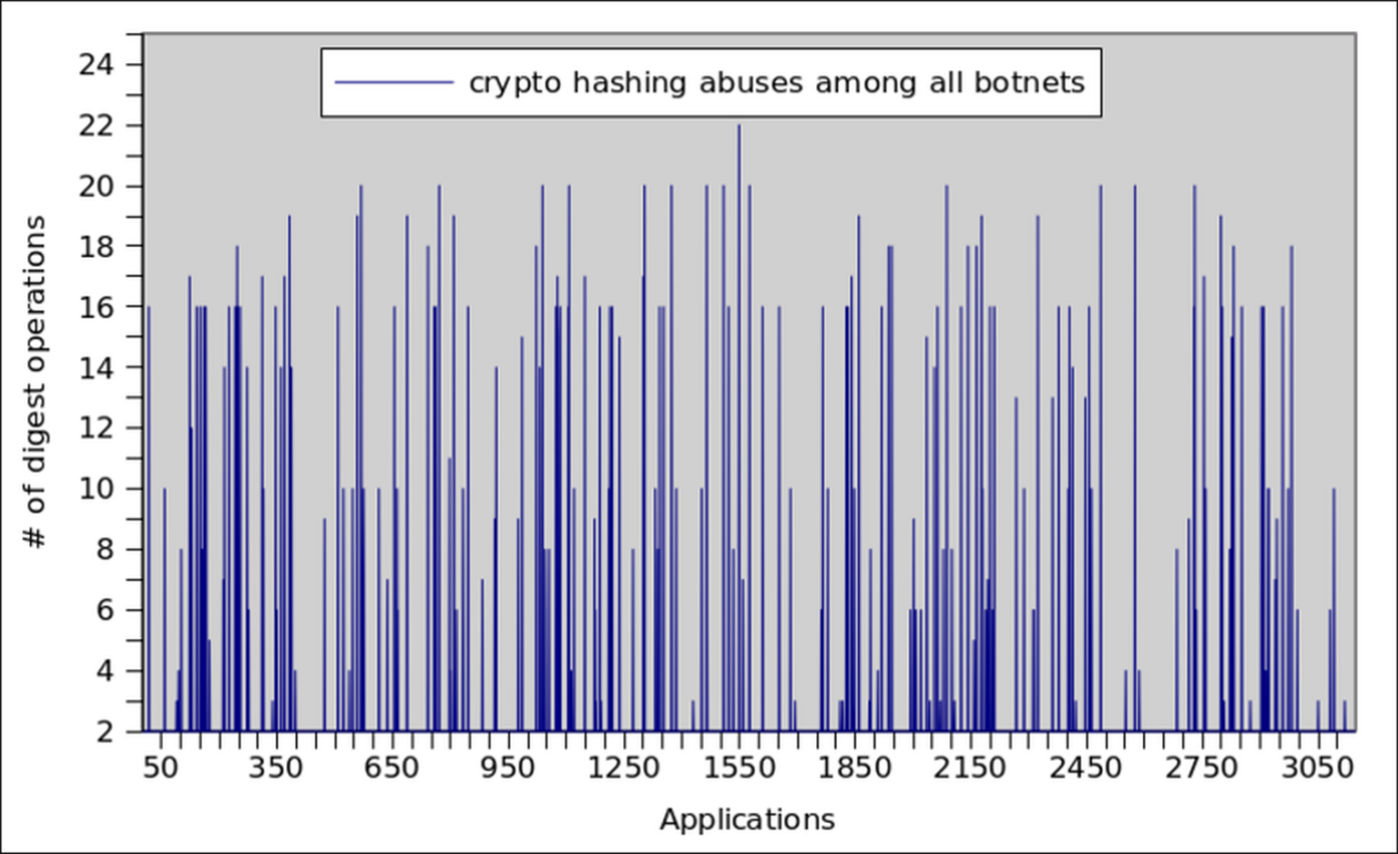
Message digest operations misused by Botnet applications.

**Fig 12:**
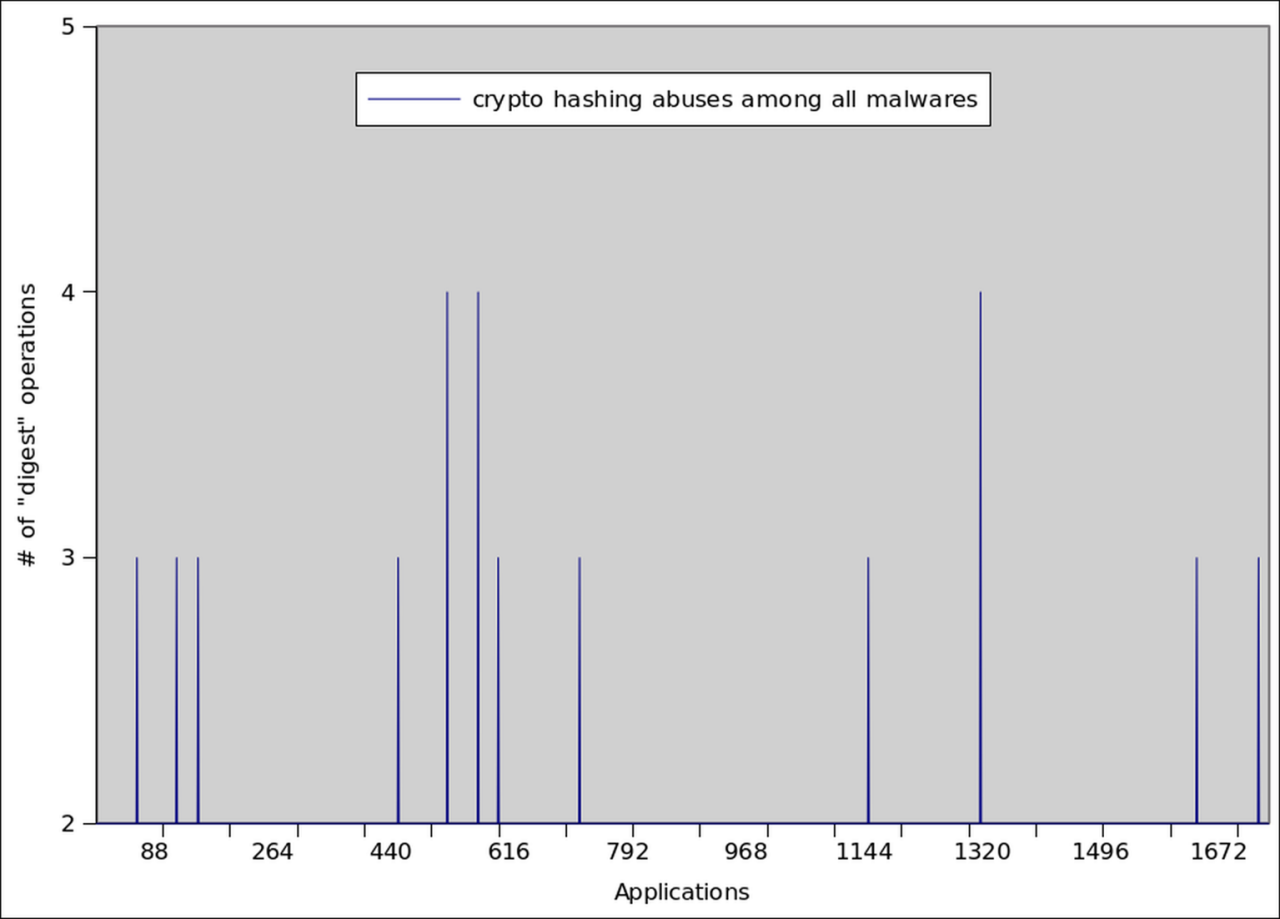
Message digest operations misused by Malware applications.

Message Digest (MD5) is a widely accepted standard for enforcing message integrity during the network communication. However, recently researcher have found some serious security concerns in the form of collision attacks [72] and replay attacks [73]. Therefore, recent studies [74] not encourage users from adopting this option. The results regarding MD5 misusage by botnet and malware applications are shown in Figs 11 and 12 respectively. We observed high spikes when digest operations were misused in a large number of botnet applications. On the average, each botnet application misused 14±2 digest operations, whereas only 12 malware samples misused 3±1 digest operation on the average.

### DNS Statistics

From the defense point of view, we must be aware of what properties exist which can distinguish botnet traffic from legitimate network traffic relying heavily on DNS protocol. For this purpose, various studies have conducted to compare DNS queries generated by botnet attack or by benign sources. As a result, according to [50,51], we can differentiate botnet and regular DNS queries by investigating (a) botnet structures (b) botnet synchronization and (c) bots response time. Another study [52] stated that various factors contribute to the malicious behavior of DNS traffic, such as (a) low time to live (TTL) values, (b) large volume of DNS query requests that indicates a botnet intention (c) number of failed DNS queries and (d) the number of responses return by the DNS server i.e DNS_TYPE_A record.

**Fig 13:**
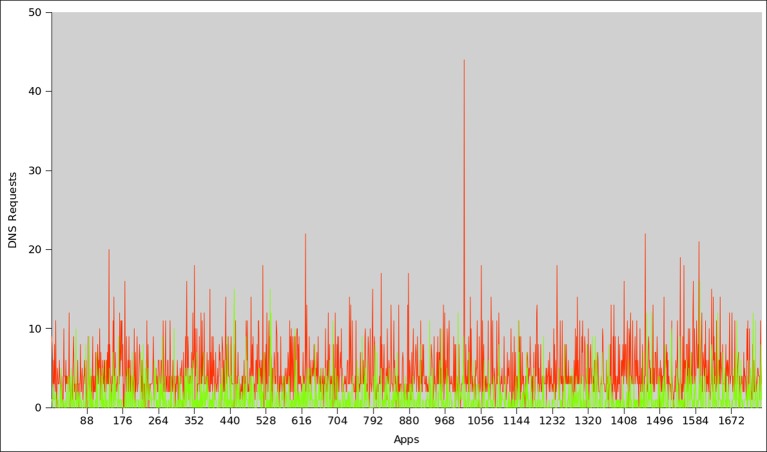
DNS Requests for botnet and malware apps.

**Fig 14:**
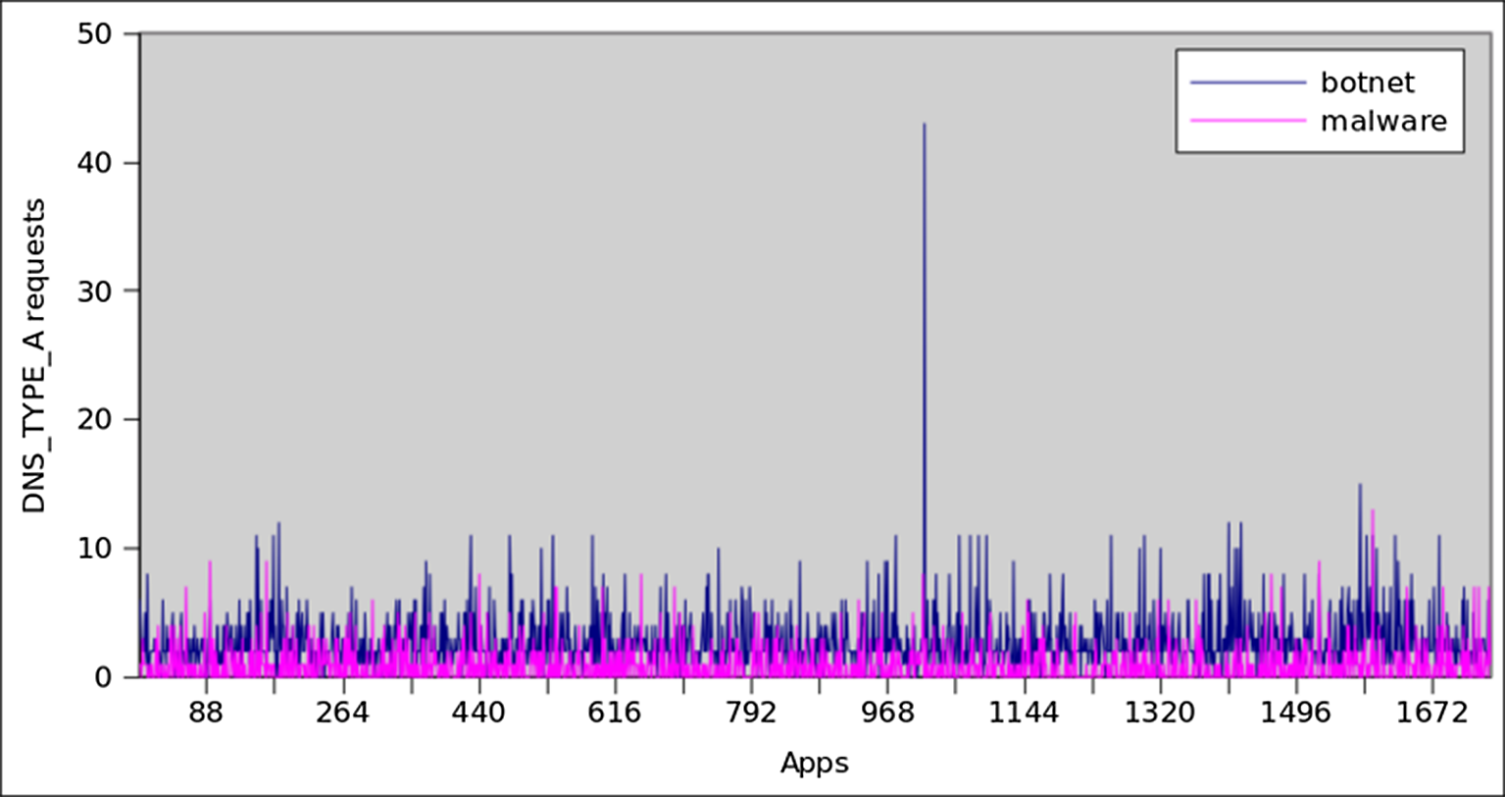
DNS_TYPE_A record comparison.

**Fig 15:**
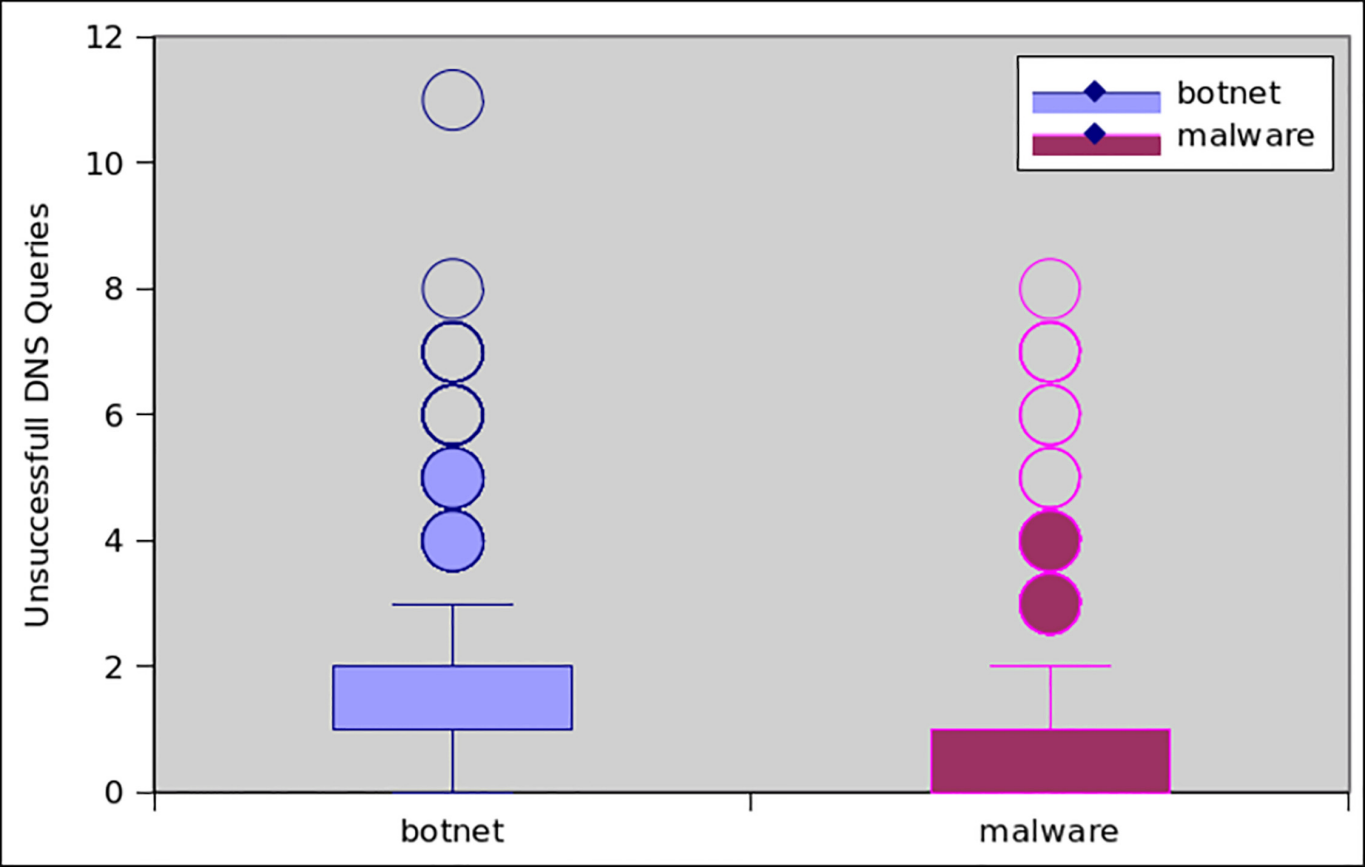
Unsuccessful DNS queries by applications.

Fig 13 shows the comparison of the top 1746 botnet and malware applications with respect to DNS requests. It clearly indicates that the botnet applications request more DNS queries than the malware applications. On the average, each botnet application requests 4.4 DNS queries, whereas on the average each malware initiates 3.1 times DNS requests. Overall 96% of botnet applications perform DNS requests, in opposite only 51% of malware samples requested DNS queries. Another important factor to assess the botnet intuition is to determine the frequency of failed DNS queries. This also affirms our classifiers’ accuracy that botnet dataset has higher failure rate with respect to DNS queries, while malware has lower rate of failed DNS requests. Consequently, 80% of the botnet applications have failed DNS requests, while only 28% of the malware samples have failed DNS requests. As for as DNS response record is concern, 95% of the botnet applications receive (average of 2.7) DNS server replies, whereas only 48% malware samples receive (average 0.9) DNS response. Fig 14 shows the response generated by DNS server also known as DNS_TYPE_A_Requests. Similarly, the total number of unsuccessful DNS queries is presented in Fig 15. On the average, the DNS server’s responses for botnet applications are more than those for malware samples. Moreover, a similar trend was observed for unsuccessful DNS queries generated by the botnet applications, i.e it is higher than malware applications.

### File Operation Statistics

Android applications can access internal storage and external storage from SD cards. According to analysis conducted by [71], overall 72% of benign application and 96% of malicious application access files for reading, whereas 83% of benign and 95% of malicious applications access file system in write mode. Botnet application can use file system activities to store malware binaries to external storage.

**Fig 16:**
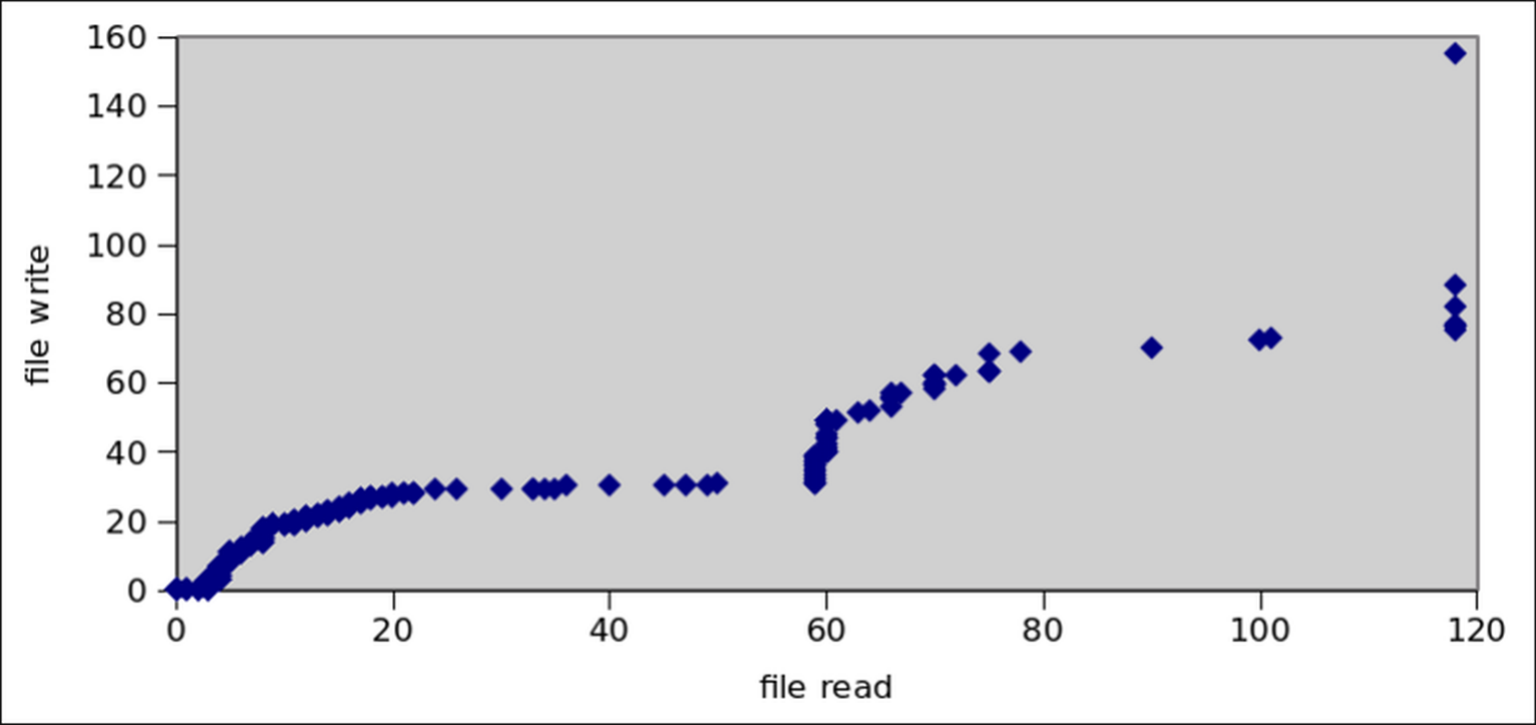
Average File Read/Write operations by malwares.

**Fig 17:**
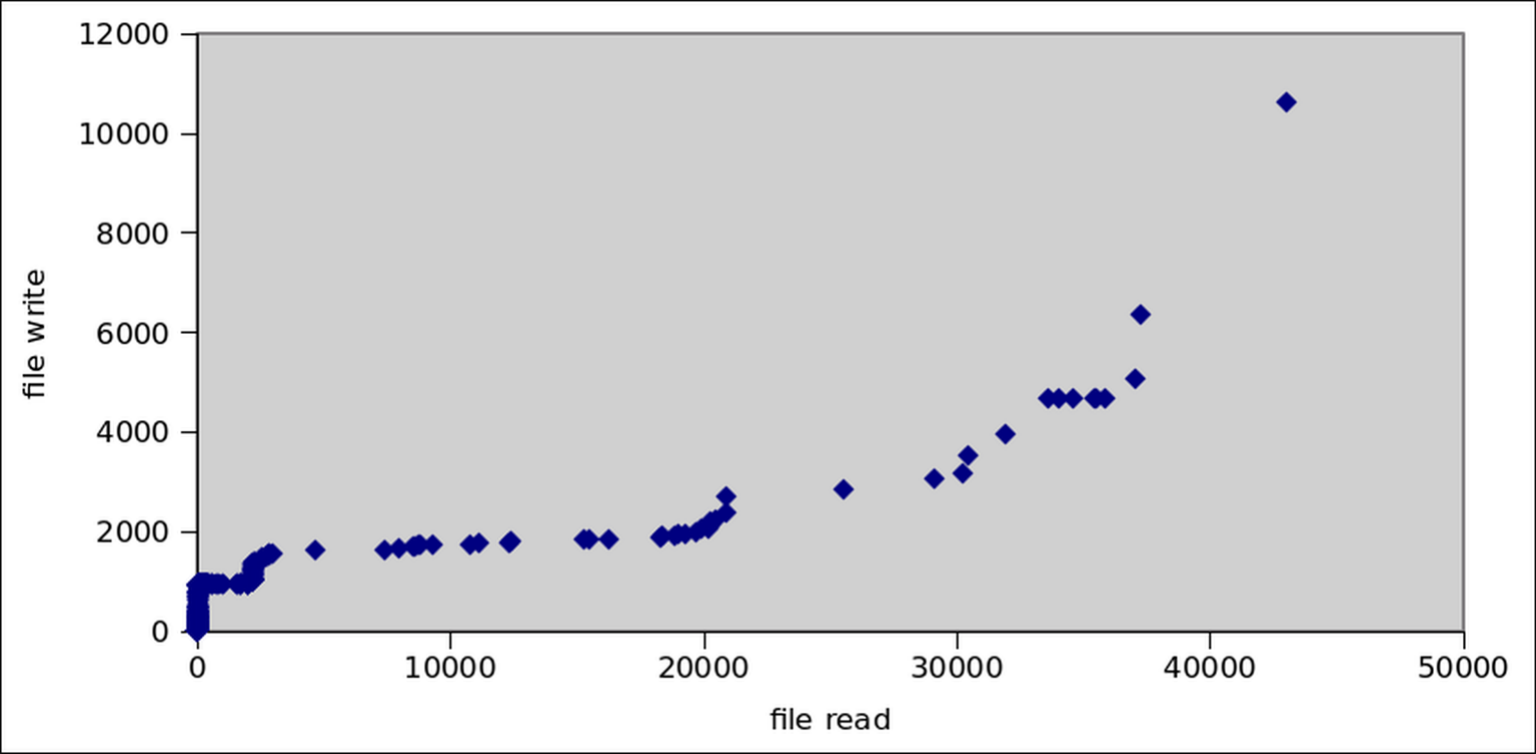
Average File Read/Write operations by botnets.

**Fig 18:**
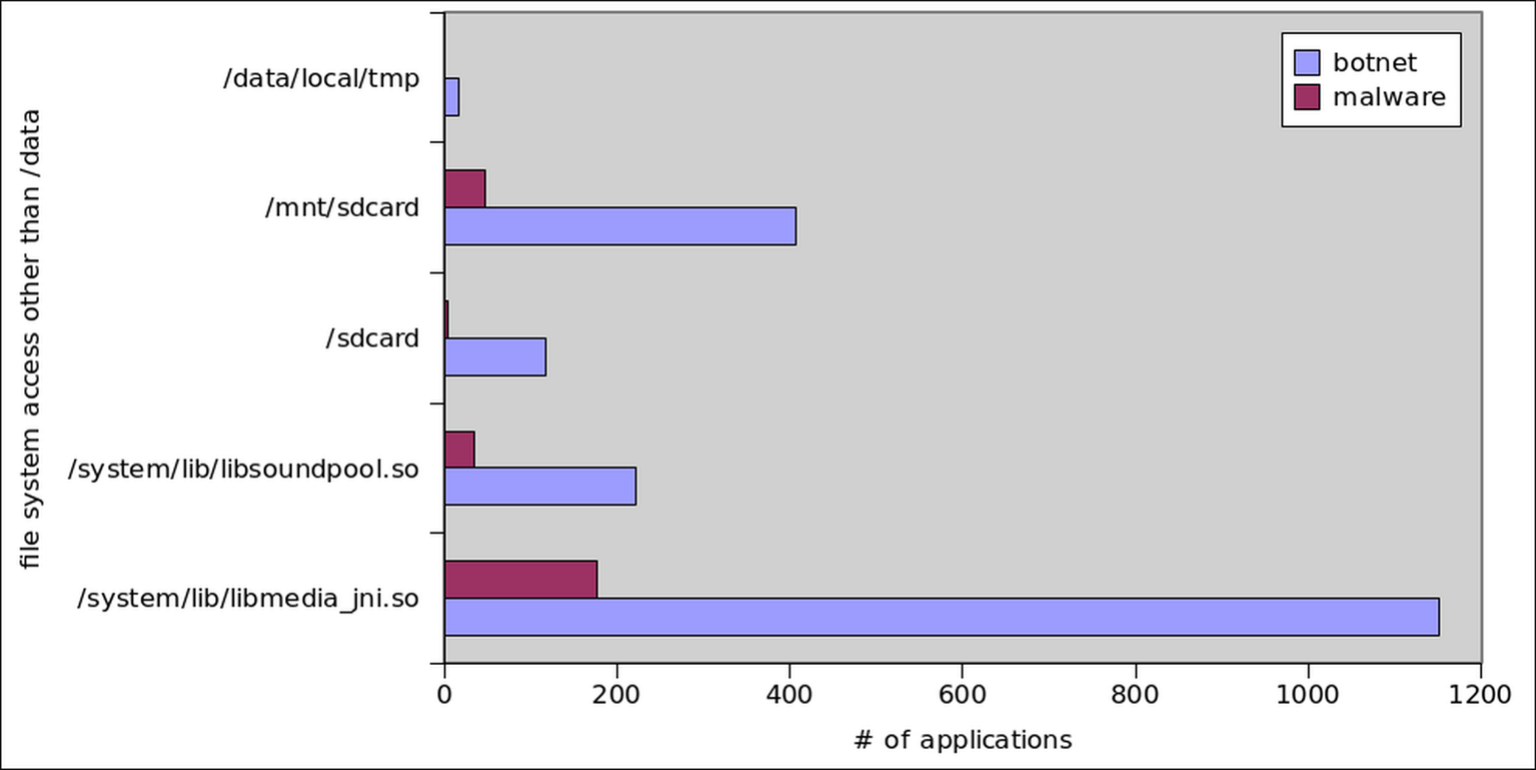
File System Access to Applications.

**Fig 19:**
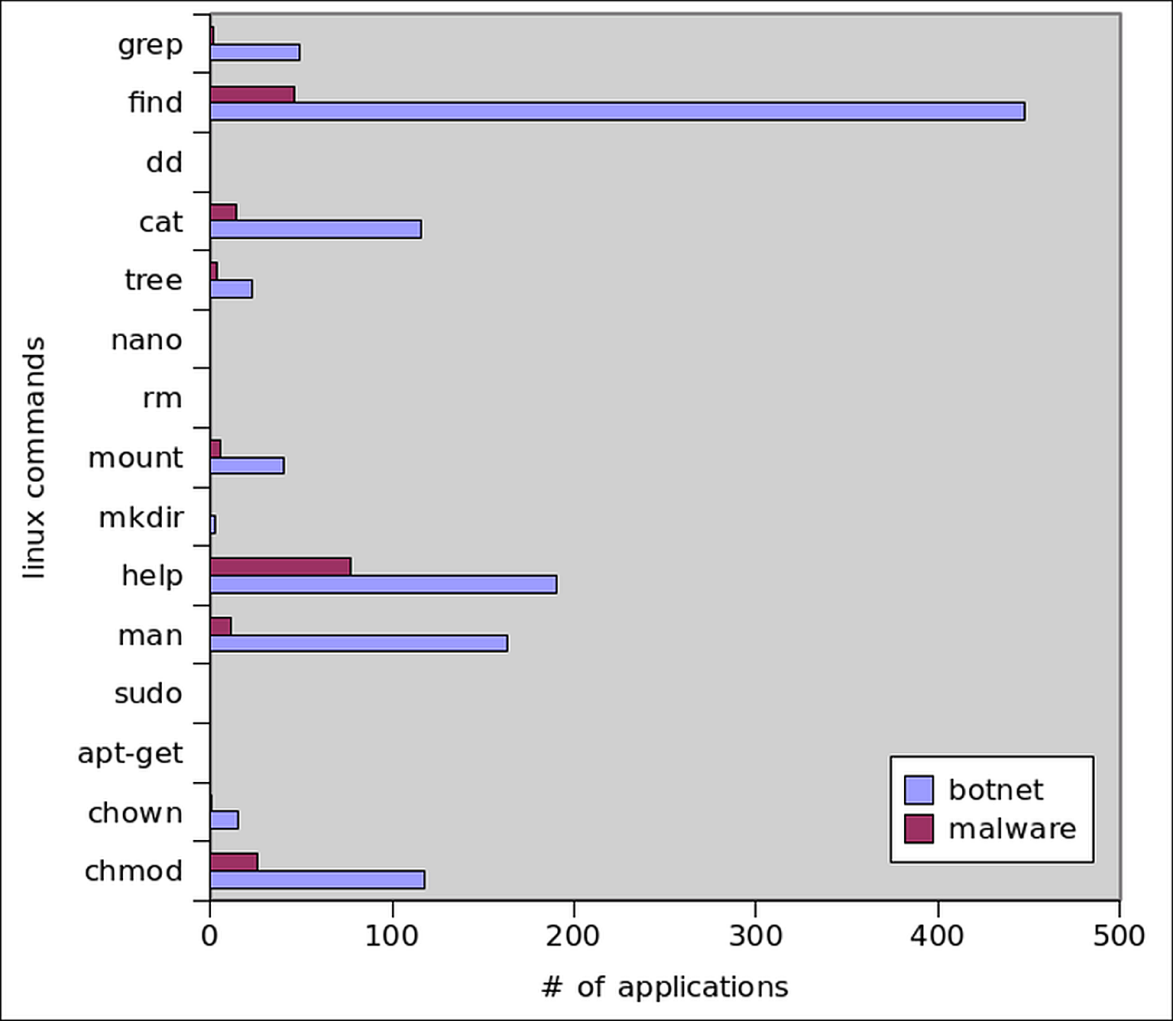
Linux Commands Usage.

Botnet applications need to access the file system of Android OS in order to store malware binaries and to interpret personal data. For this purpose, apps extensively utilize internal and external storage from SD cards. Figs 16 and 17 show the read and write operations’ frequency in all malware and botnet applications respectively. We observed that, on the average read and write operations performed by botnet applications are predominantly higher at 404.25 and 208.28 respectively, whereas the trend for reading/writing operation in malware applications is extensively low at 6.66 and 5.60 respectively. Moreover, as for as the file system access to primary storage (/sdcard) and access to secondary storage (/mnt/sdcard) is concern, the Fig 18 apparently shows that access to secondary storage is far more prevalent in botnets: 12% of botnet applications access SD card, while 2% malware applications access secondary storage. Moreover, the primary storage is 3% utilized by botnet applications whereas malware application used only 0.2% access to primary storage of Android systems. Furthermore, access to shared media libraries (libmedia_jni.so and libsoundpool.so) is also highly recommended by botnets. Moreover, we also gathered the statistics regarding the applications intended to call root level Linux system commands which are presented in Fig 19. We concluded that root level access commands like *chmod*, *chown* and mount has higher frequency of calling in botnets than in malware. The reason is simple that is to get controlled over the device. Similarly, the commands related to content searching like find, cat, grep, help, and man are also desirable for botnet applications. As a sum, *chmod*, *chown*, *mount*, *find*, *man*, *help*, *grep* and *cat* commands are used by 26,1,6,46,11,77,2, and 14 malware applications respectively. In contrast, for botnet dataset 118, 15, 40, 448, 163, 190, 49, 116 applications used the above mentioned commands.

### HTTP Statistics

Smartphones are connected to the Internet, and the C&C functionality for Android botnets is constantly controlled through the network. In consequence, bots in a mobile botnet are enforced to receive control server commands through Hypertext Transfer Protocol (HTTP). Despite the fact that INTERNET permission is explicitly utilized when an application has to obtain network access, this permission is considered common and cannot be the only identifier for malicious behavior of an application. One of the popular example for HTTP based botnet is Geinimi botnet [46], which enables encrypted communication between C&C server and bots with the help of DES encryption scheme and via legitimate HTTP POST requests. Moreover, Geinimi also used DES to encrypt domain names of C&C servers. Hence, it was proven as the most sophisticated botnet that times [53].

**Fig 20:**
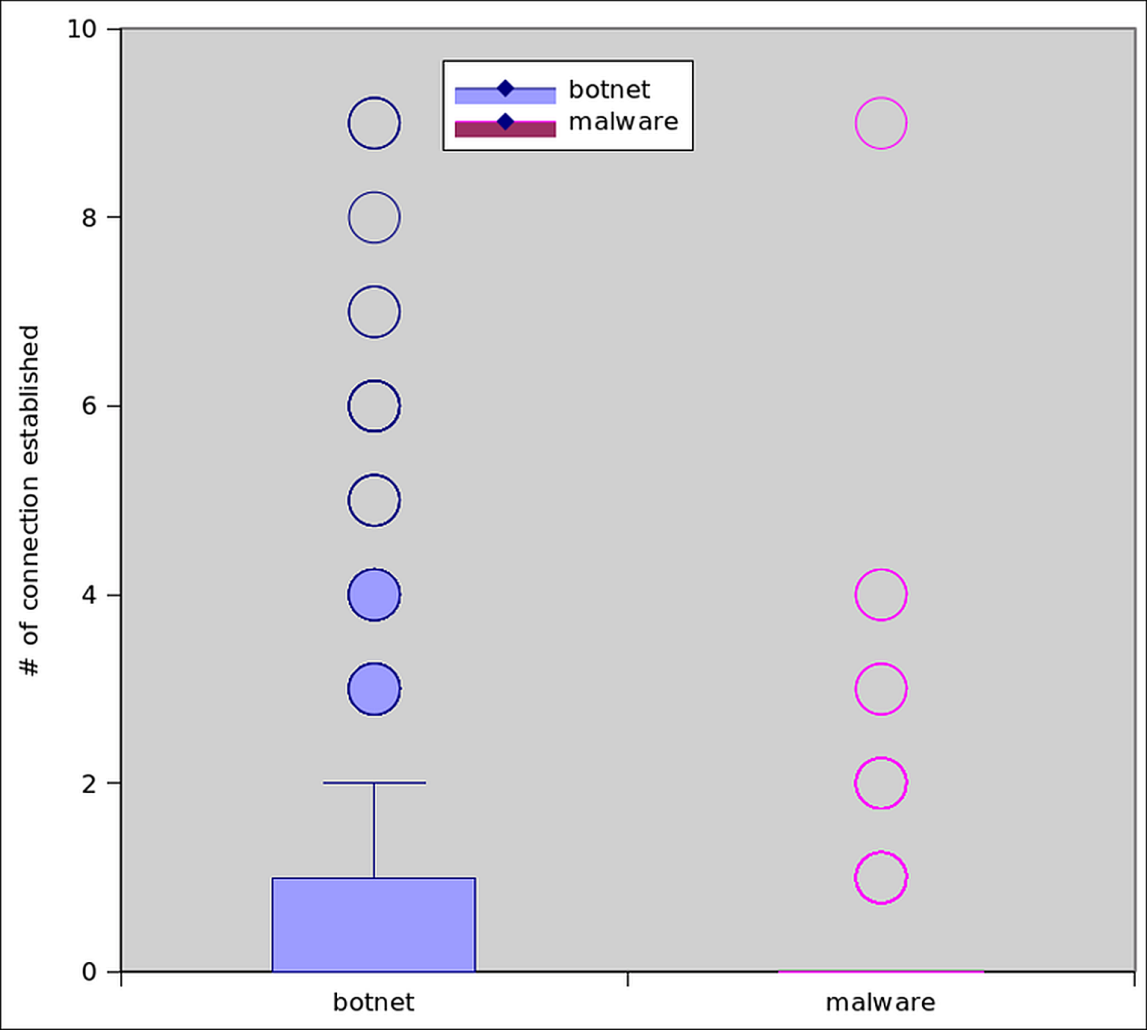
Total Number of Established Connections.

**Fig 21:**
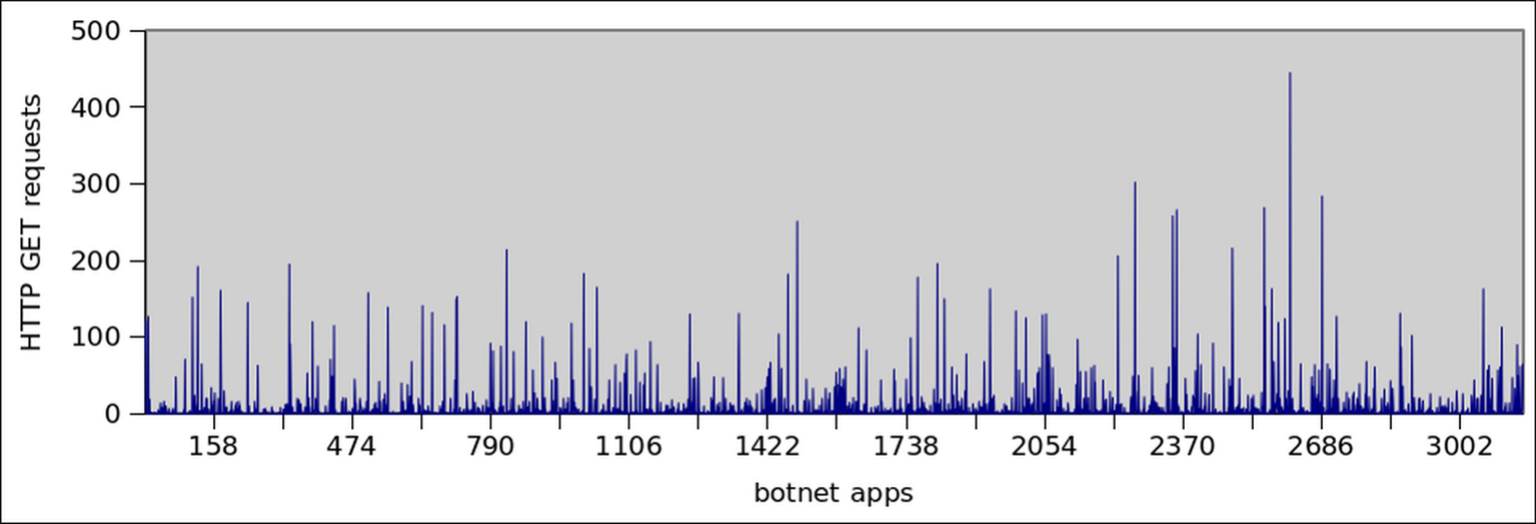
HTTP Get Requests by botnet Applications.

**Fig 22:**
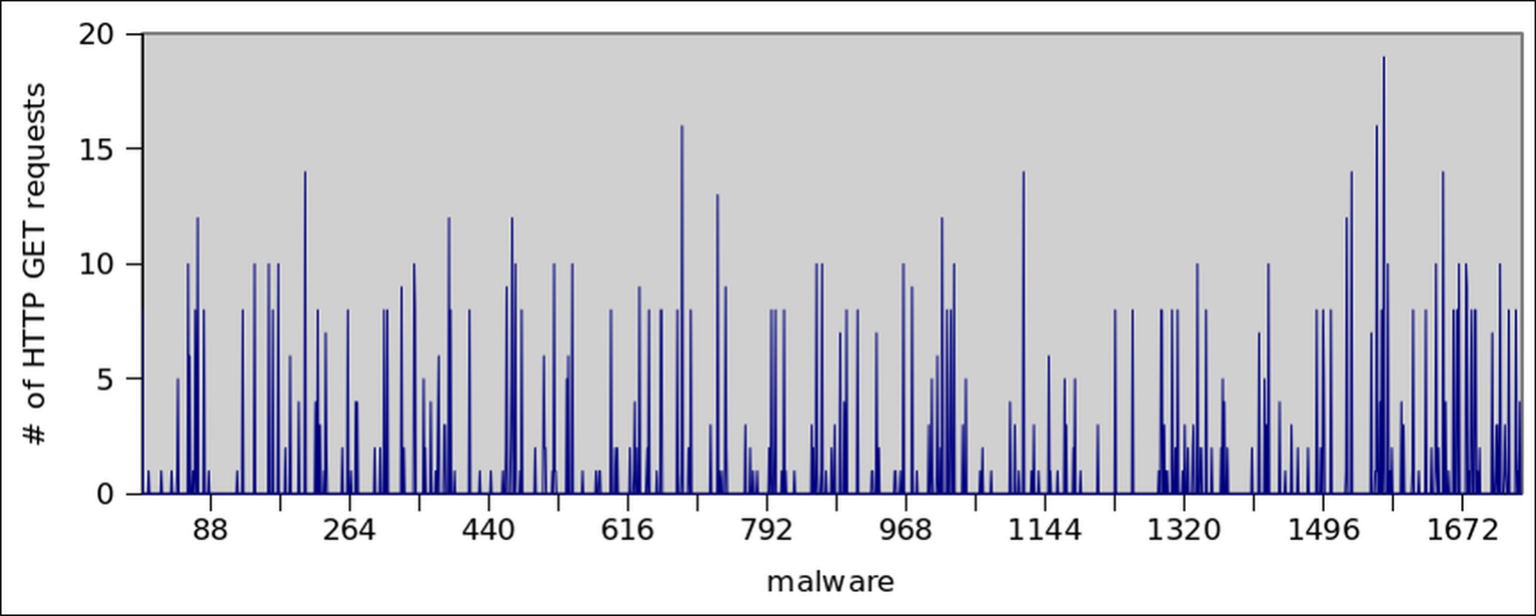
HTTP Get Requests by Malware Applications.

HTTP protocol governs communication in two ways, by GET or POST request. A GET request is meant to retrieve static contents like images, binaries etc. while POST requests are used in server side programming to dynamically retrieve the resources. Thus, HTTP attacks generated by GET requests are simpler to create, and can more effectively scales in a botnet scenario [54]. Fig 20 shows the number of HTTP connections opened/established by botnet dataset. It clearly shows that, 92% of botnet dataset established TCP connection, whereas only 33% malware do so. Moreover, the average connections established by each botnet and malware application are 10 and 2.5 respectively. In order to get insight into the HTTP traffic, we also observed the GET requests initiated by both datasets. It can be seen from the Figs 21 and 22 that 40% of the botnet applications use GET command for communication, however, 23% of the malware samples use this feature to communicate externally via HTTP.

### Data Leak Statistics

**Fig 23:**
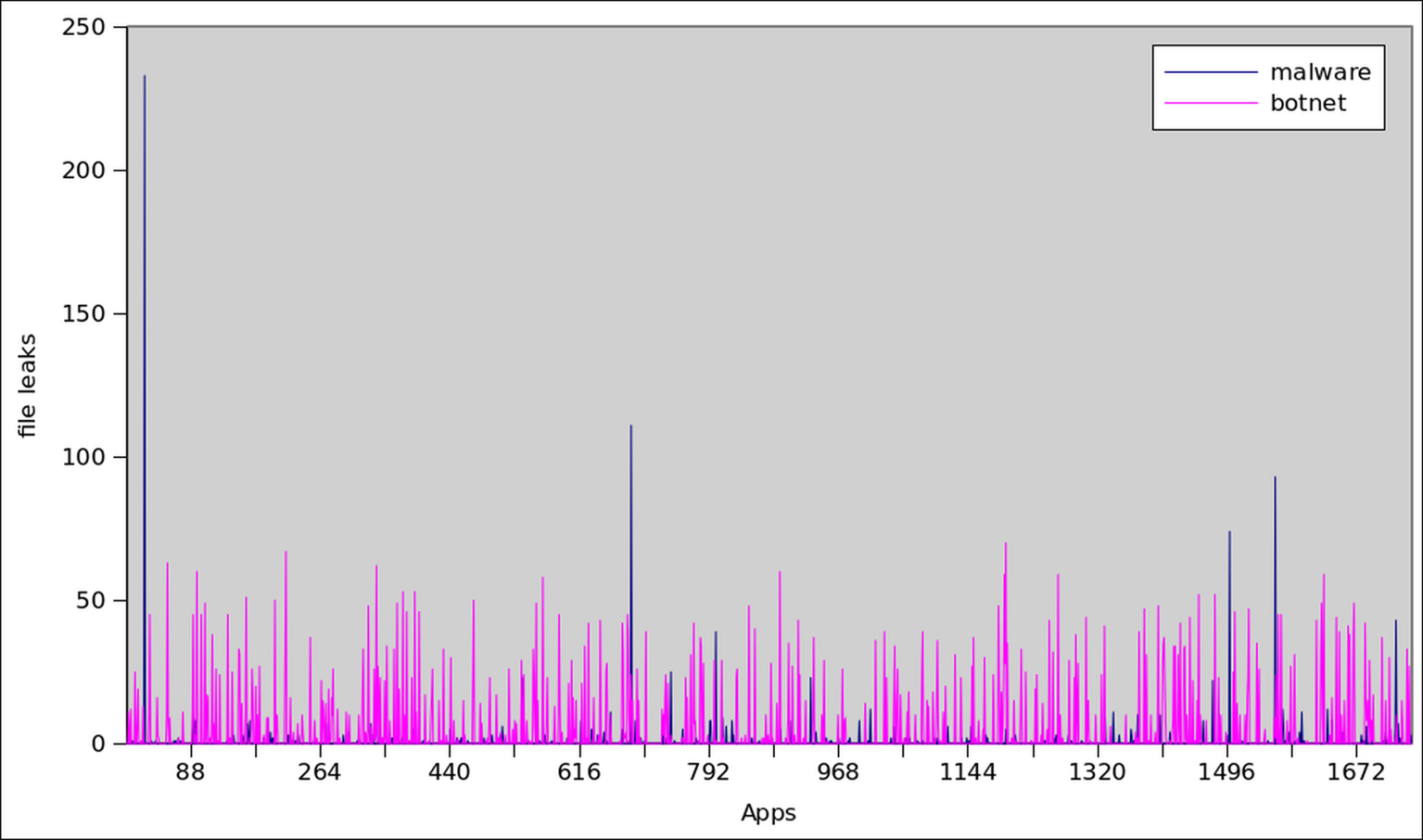
File Leak statistics among all Botnet and Malware.

Data leakage is considerably more frequent among botnet than malware: overall, 26% of botnet applications leak user information over the internet, whereas 11% of malware applications do so. However, botnet and malware applications leak device-specific information, for instance, International Mobile Subscriber Identity (IMSI), International Mobile Station Equipment Identity (IMEI), Integrated Circuit Card Identifier (ICCID) and subscribers’ phone number. Overall, 44% and 23% botnets leaks the IMEI and phone number respectively, whereas only 9% of malware leaks ICCID. Similarly, it is common in botnet samples to leak names and phone numbers from the subscribers’ address book and disseminate this information to their C&C. Whereas, leakage of names and phone number is uncommon factor observed in malware dataset. Fig 23 shows the trends of file leakage in top 1745 botnet and malware samples.

### Network Operation Statistics

**Fig 24:**
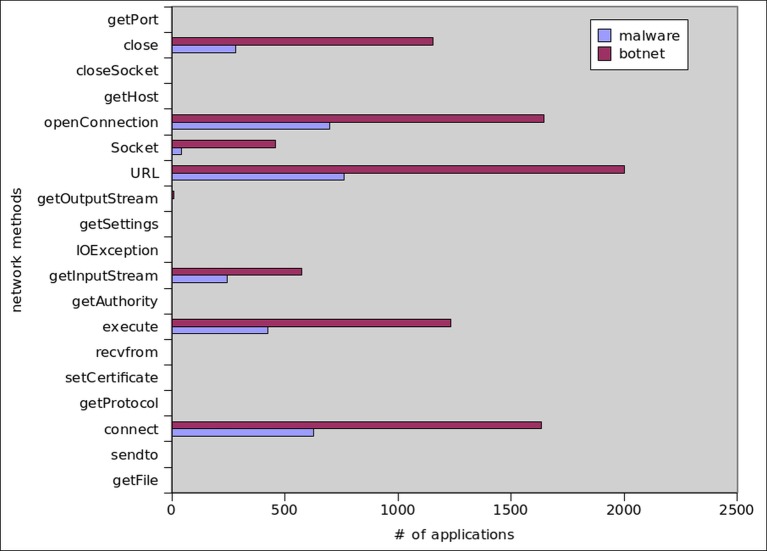
Most Common Network Methods Called by Botnet and Malware Applications.

Mobile botnet applications rely not only on permissions but also on different API functions, such as *Connect*, *openConnection*, *execute*, *URL* and *Socket* etc. We also observed these methods in our behavioral analysis system. The results in Fig 24 affirm that botnets are more interested in using these network methods than malware. Most popular network methods among botnet samples are, *connect*, *execute*, *getInputStream*, *URL*, *Socket*, *openConnection* and *Close* commands which are called by 52%, 39%, 18% 64%, 15%, 52% and 37% of the botnet applications respectively. In opposite, 36%, 24%, 14%, 44%, 2%, 40% and 16% of malware samples utilized above mentioned commands respectively,.

**Fig 25:**
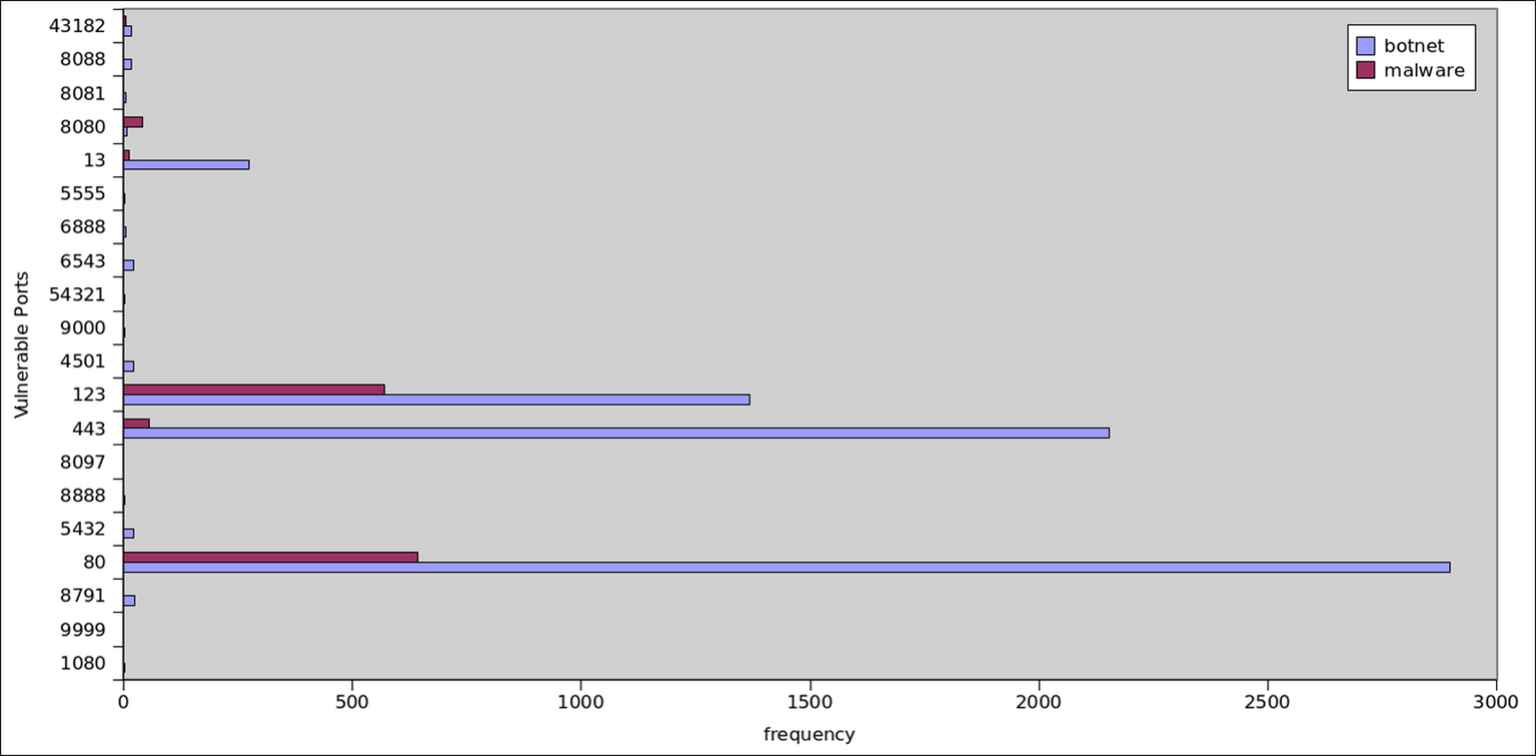
Vulnerable Ports Analysis between botnet and malware applications.

From the Fig 25 we can conclude that, the most commonly observed opened network connections for botnet dataset occurred on port 80 (HTTP, 92% of samples), port 443 (HTTPS, 69%), port 123 (NTP, 44%) and port 13 (Daytime, 9%). Whereas, 8088(HTTP), 8080 (HTTP), 6888 (P2P), 6543 (lds-distrib), and 5432 (postgresql) with less than 1% of applications each. However, for malware we observed 80 (HTTP, 37%), 443(HTTPS, 3%), and 123 (NTP, 33%).

### Services Statistics

Android applications use service model to initiate background services. Service model does not provide graphic interface to configure (similar to as Activity model) rather they are designed to offer background functionality of an applications. Malware authors use this model to establish communication pathway with Command and Control (C&C) servers of botnets, steal personal information or forward contact information to an adversary without user information.

**Fig 26:**
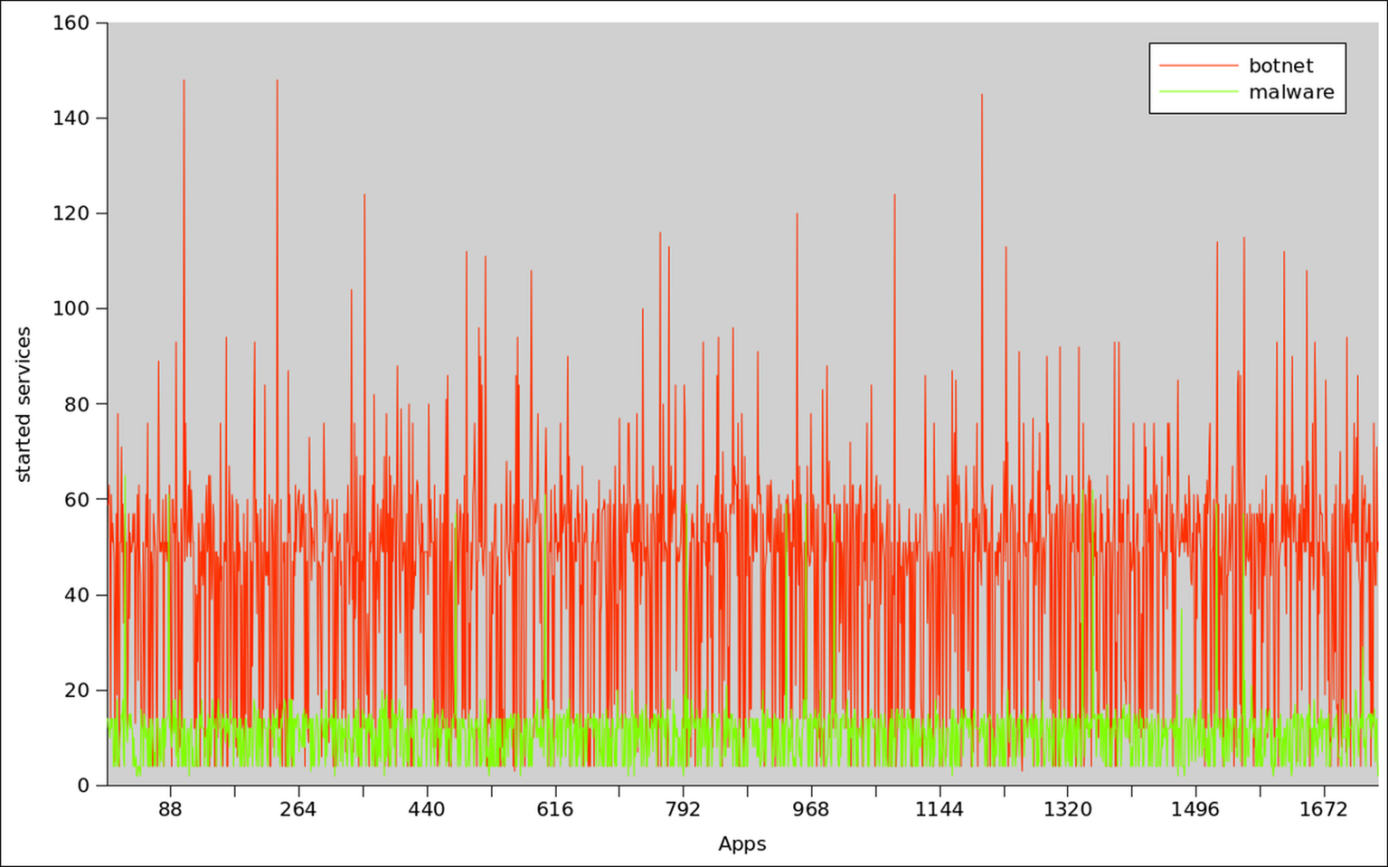
Started Services frequency analysis between botnet and malware applications.

As we described earlier, botnets initiate large number of services as compared to benign or malware applications. Previous researchers [27] reported that Android malware usually request for more services, permissions and receiver components as compared to benign applications. This behavior is attributed to, the attempts of Android malware to hide malicious actions while inaudibly executing more background services. A recent report by Forensiq [75] states that mobile botnets are costing advertisers $1 billion in ad fraud by loading bulk of advertisements. The process is carried out through loading far more ads than any benign application would—more than 20 ads per minute. In many cases the ad events are generated when the applications are not being interacted i.e. by enabling various background services/processes. Therefore, the same results reflected in our observation shown in Fig 26 that botnet applications require more services to initiate as compared to malware ones. On average, botnet applications requested 58±10 background services, whereas on the average malware applications calls background services 15±2 times.

### SMS Statistics

**Fig 27:**
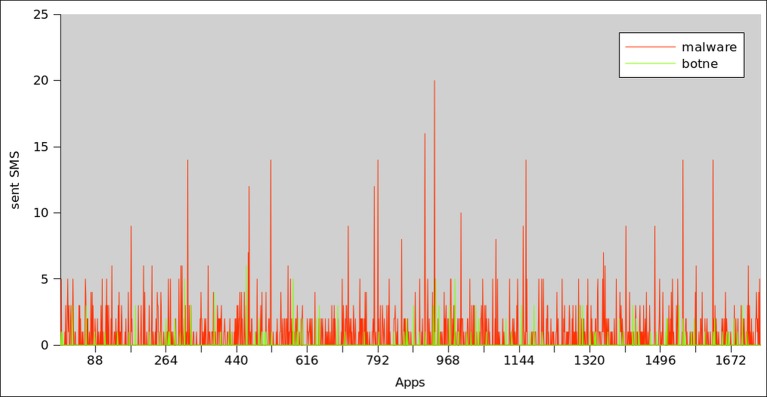
Sent SMS frequency Analysis between botnet and malware applications.

A common activity that needs to be investigated during runtime analysis of mobile applications is the frequency of sending SMS. From the Fig 27, we observe that, the percentage of sending SMS messages is higher in malware samples i.e 35% of malware apps called SENT_SMS permission. While the percentage of sending SMS in the botnet dataset is only 5%, which could be explained by the following reasons: (a) our training dataset contains 80% of botnet applications having HTTP communication protocol. (b) Sending messages to a premium rated number is a popular technique used by mobile malware [57]. Hence, the results affirm our classifier’s accuracy by showing the high sent SMS frequency rate in the malware dataset.

## Performance Evaluation

In this section, we present various performance measures to recognize the degree of effectiveness of our SMARTbot framework. We make comparison with respect to model efficacy, scalability and performance comparison. Furthermore in this section, we provide a case study that helps to demonstrate the usability of our framework.

### Model Efficacy

To measure the reliability of our classifier, we further applied random sampling method to our selected datasets. For random sampling, we assigned 66% training data instances and 33% for test dataset. Although, we obtained similar results while choosing the best option between cross validation and random sampling, yet 10-fold cross validation generates slightly better results as compared to random sampling. The results in Table 6 affirm the viability of the simple logistic regression classifier as a basis for effective botnet application detection within the specified feature domain. Ultimately, this will become our final choice for classifier building in production environments.

**Table 6:** Model Comparison between 10-fold and random sampling.

	10-Fold Cross Validation								Random Split Validation (66% Training, 33% Testing)							
	Accuracy	Precision	Recall	F-Measure	TPR	FPR	TNR	FNR	Accuracy	Precision	Recall	F-Measure	TPR	FPR	TNR	FNR
Naive Bayes	90.76	0.87	0.87	0.87	0.87	0.07	0.93	0.13	90.20	0.86	0.86	0.86	0.86	0.08	0.92	0.14
SVM	95.58	1.00	0.88	0.93	0.88	0.00	1.00	0.12	94.23	1.00	0.84	0.91	0.84	0.00	1.00	0.16
MLP	97.10	0.94	0.99	0.96	0.99	0.04	0.96	0.01	97.41	0.95	0.98	0.96	0.98	0.03	0.97	0.02
simple logistic regression	**99.49**	**0.99**	**1.00**	**0.99**	**1.00**	**0.01**	**0.99**	**0.00**	**99.46**	**0.99**	**0.99**	**0.99**	**0.99**	**0.01**	**0.99**	**0.01**
J48	98.14	0.98	0.97	0.97	0.97	0.01	0.99	0.03	97.84	0.97	0.97	0.97	0.97	0.02	0.98	0.03
RF	98.83	0.99	0.98	0.98	0.98	0.01	0.99	0.02	98.38	0.98	0.98	0.98	0.98	0.01	0.99	0.02

In addition to that, we can implement this classifier model on user devices in order to predict the granularity of botnet behavior in running applications. As our future work to implement this model in mobile apps it will make users able to predict correct class of application with the help of observed behavior. Similarly, Table 6 depicts the learning time comparison between 10-fold cross validation and random sampling. Training time is ranging from 1.9957s to 3.5611s in 10-fold cross validation whereas random sampling requires 0.036s to 8.076s to train the model. Additionally, testing time required by 10-fold cross validation ranges from 0.0321s to 0.0691s which is better than existing machine learning based mobile malware detection solution, Mobile-Sandbox [76]. Likewise, the time taken to process testing classifier model during random sampling is 0.018s to 3.90s. Moreover, Table 7 shows the size of each classifier in order to measure the feasibility to deploy it to user device. We observe the same model size in both 10-fold and random sample scenarios. However, the largest size for any model is 1.6MB which is of the simple logistic regression model. In contrast to MLP and Naive Bayes model sizes in [77], our model size is reasonable enough to reside on user device.

**Table 7:** Time and Size Comparison.

	10-Fold Cross Validation				Random Sampling			
	TrainingTime(seconds)	Testing Time(seconds)	Model Building Time(seconds)	Size (KB)	Training Time(seconds)	Testing Time(seconds)	Model Building Time (seconds)	Size (KB)
**Naive Bayes**	3.5611	0.0691	0.02	8	0.941	0.095	0.02	8
**SVM**	1.9957	0.0544	8.87	479	6.973	3.906	8.85	479
**MLP**	3.0953	0.0321	10.54	25	7.21	0.296	10.7	25
**simple logistic regression**	3.2538	0.0541	5.4	1598	8.076	0.018	5.36	1598
**J48**	2.3205	0.0613	0.09	23	0.036	0.026	0.18	23
**RF**	3.0645	0.0328	1.56	1359	0.99	0.06	1.61	1359

### Scalability

To date, the majority of proposed solutions can work either as on-device or off-device analysis systems which result in scalability issues. However, we look the scalability of the SMARTbot framework from both perspectives: when this solution is deployed to large-scale market stores as an offline analysis option and when embedding the classifier into user device for the runtime analysis of installed applications. At present, we can deploy SMARTbot as an offline analysis framework to large-scale market places (e.g Google Play) without much effort. We calculated the time required to generate a mobile botnet classifier model. As a result, the total time required to generate report links for the Drebin dataset (4891 malware binaries) is ≈20 hours which include, the uploading time, loading time to the sandbox, execution time, report generation time and network communication overhead from cloud to the host machine. On the average, each application requires almost 20±5 minutes to completely execute and generate log reports. Here, we need to highlight that, although this is an ideal elapsed time to process an application in sandbox, yet it is not obvious in all cases. There are many factors that contribute to the extension of processing time, e.g. system’s peak hour, temporary disruption of service, network communication outage. In this study, the feature extraction time is approximately 15 minutes for the execution of Python parsers and collection of values against feature vector. However, machine learning-based classifier only consumes a few seconds during the testing phase to predict the class of an application.

The deployment of SMARTbot logic directly into smartphone devices requires the design and development of an Android application to support our machine-learning classifier; such an application will be part of our future work. Thus, we conclude that, our framework that applies behavioral observation is feasible for hundreds or even thousands of applications.

### Performance Comparison

In this subsection, we compare SMARTbot framework with existing related approaches to highlight the significance of our work. Exiting approaches employ static, dynamic or hybrid approaches with varying dataset sizes and focus on general malware detection; therefore, direct comparison is not feasible. However, we can compare the classification results in terms of machine learning techniques employed, accuracy and precision.

The hybrid behavioral model proposed by [78] employs an SVM classifier for training and testing purposes and achieves 96.9% accuracy. For this model, a dataset of 3368 malicious applications was used for classification. Another work selected for comparison is [79], which is also based on static analysis. It uses Permission and API calls as the feature vector and evaluates the results with various machine learning approaches such as SVM, Bagging and C4.5. For comparison, we selected the best results obtained by the model using the SVM classifier and achieved 96.69% accuracy. In addition, authors in [77] proposed an Android malware detection system using Bayesian algorithm with static feature set including permissions and API calls. The authors conducted experiments on 1000 malware samples with various module constructions and achieved 98% accuracy for 15M-based classifier model. Another important work that we considered for comparison is [21] which produces a set of 5560 malicious applications. It uses vast and diverse array of static features as a feature vector and perform classification using SVM. This approach shows comparatively good results in terms of accuracy, (i.e. 98.78%).

**Fig 28:**
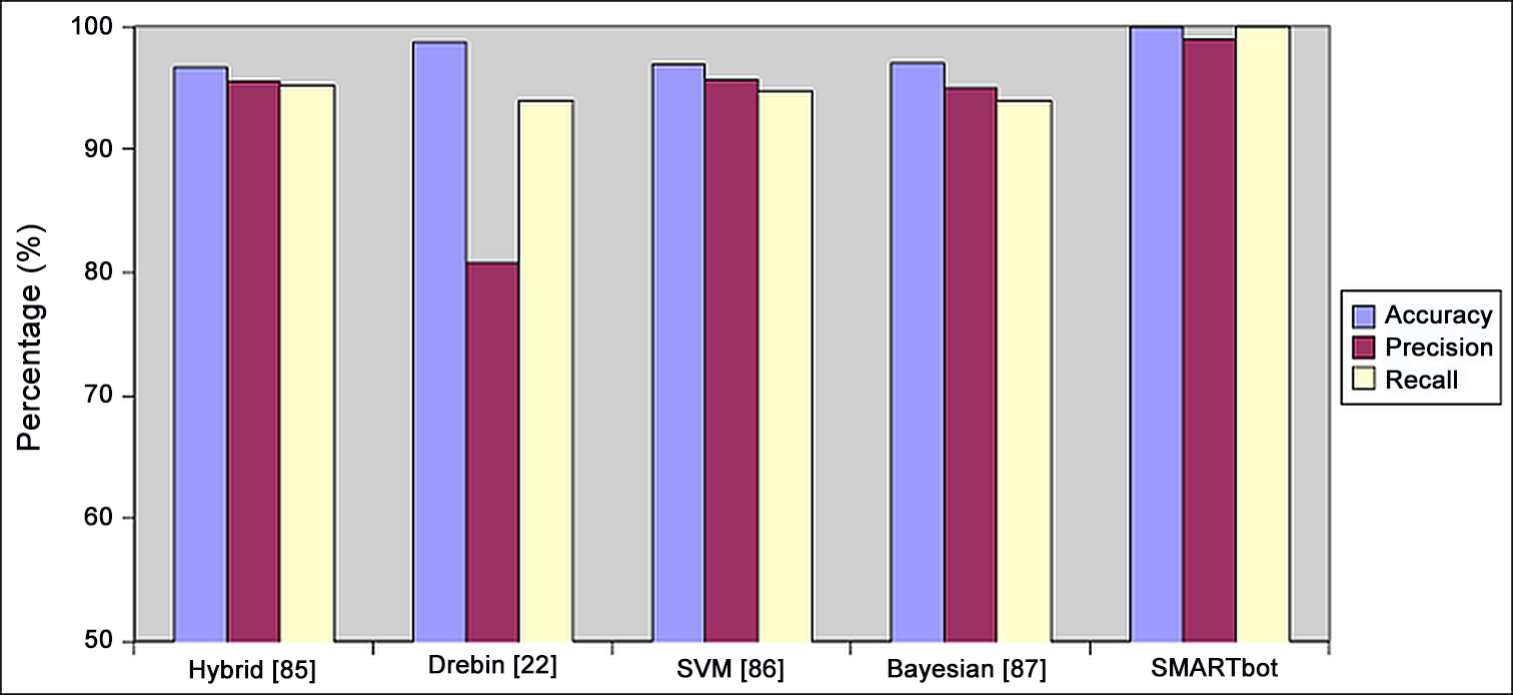
Performance Comparison with existing approaches.

On the other hand, our proposed framework SMARbot uses 4891 malware samples obtained from [21] and employs various machine learning algorithms for classification. Unlike the aforementioned approaches, SMARTbot uses dynamic analysis in order to detect botnet behavioral patterns in mobile applications. Moreover, ANN’s backpropagation modeling is used to train and label the botnet dataset. We also evaluated our model with 10-fold cross validation and random sampling and obtained better results from the 10-fold cross validation. As a result, the simple logistic regression achieves 99.49% accuracy which is comparatively better than previous approaches. Fig 28 shows the comparative analysis of SMARTbot and existing approaches.

### Case Study: Observation of Botnet Capable Suspicious Applications

In order to show the efficacy of SMARTbot, we conducted a detailed analysis of one of the existing Android based mobile botnet application NioServ (a web-proxy bot) which was introduced in May 2015 [80]. The application belongs to drive–by-download category and is not flagged as suspicious by Andrubis with malicious score 0.3/10. Unlike Andrubis, SMARTbot classified this applications as botnet.

**Fig 29:**
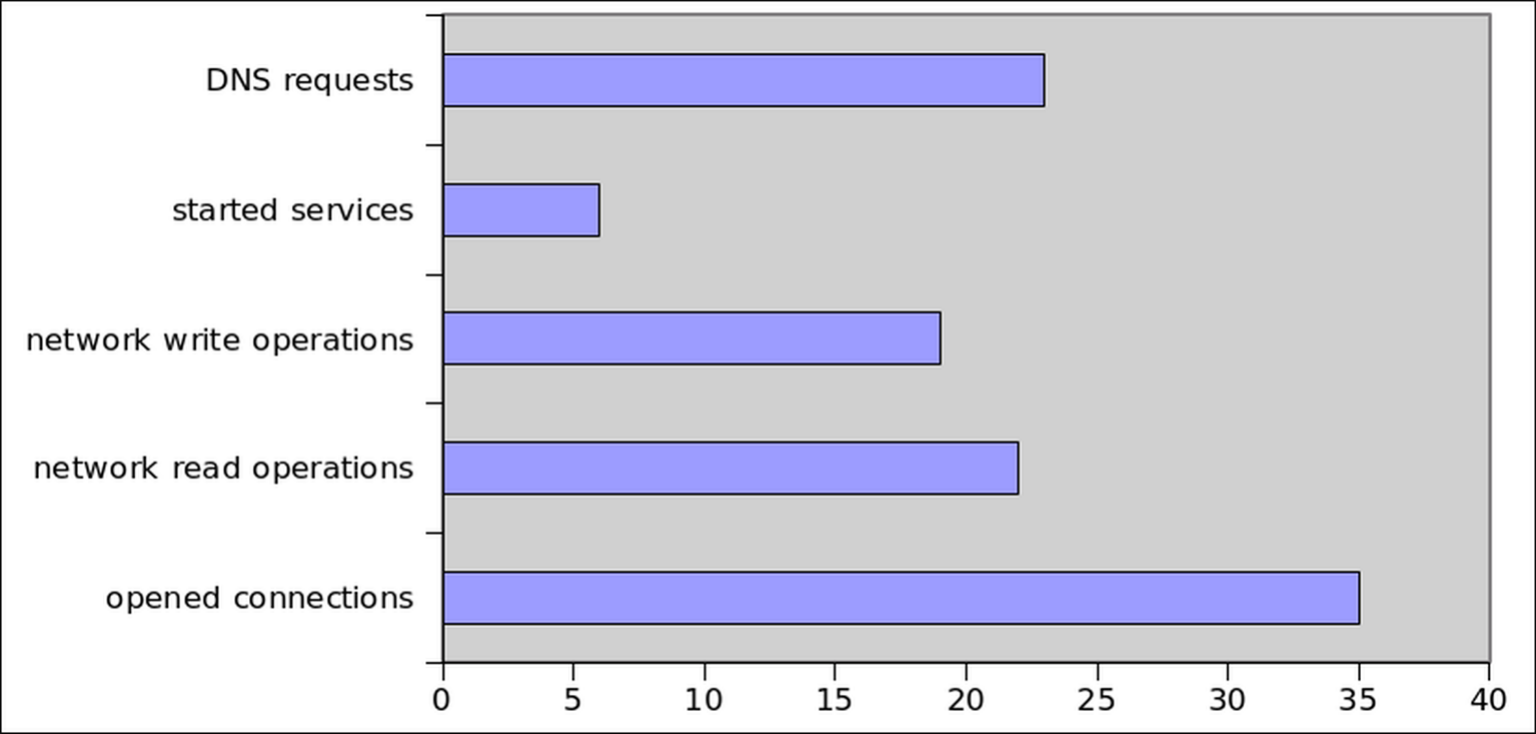
Dynamic Analysis Results of NioServ.

We manually observed static and dynamic analysis properties and found several interesting facts about this application. During network traffic analysis, the application attempted to connect to remote hosts with 35 distinct IP addresses. Moreover, a TCP session with a C&C server with IP 212.7.197.220 which is backlisted by [81] as spam server, was observed. In order to communicate securely, the application established connections on port 443. Fig 29 shows the number of DNS requests, background services, network read/write operations and opened network connections.

According to the static observations, the application has enabled access to INTERNET, ACCESS_NETWORK_STATE and RECEIVE_BOOT_COMPLETED permissions. With, these permissions, the bind method of Java ServerSocket class is called in order to communicate outside by opening a socket. Moreover, the application listens for RECEIVE_BOOT_COMPLETED permission to start any background activity in order to trace the smartphone location and to listen to a C&C server.

Overall, the decision of SMARTbot to mark this applications as botnet is based on the following factors: large number of network operations, including network read/write operations; large number of DNS queries; IP address of the C&C server; unsuccessful DNS queries; and numerous HTTPS-based opened connections. We conclude that using static analysis-based malware detection systems solely is insufficient to establish correct decisions; for a thorough investigation the behavioral and communication patterns of applications must be observed.

### Limitations

Although SMARTbot can effectively identify botnet specific Android applications yet it has few limitations. First the file size limit is 8MB which is inherited from Andrubis. However, we cope with this limitation by devising our own mobile sandbox with rich UI support. In addition to that, the service availability constraints of Andrubis are also present even when the service is unavailable, disrupted or malfunctioning. Second, the use of sandboxing technique is another limitation; various approaches [82] have been introduced by the researchers to determine if the execution platform is a sandbox machine or a real device. For instance, Obad botnet tries to evade execution on several sandboxes using anti-decompilation or anti-emulation approaches. It does so by checking the value of Android.os.build.MODEL, if the value indicates the existence of emulator, the application stops execution immediately [7,83].

Lastly, dynamic analysis itself requires a comprehensive set of execution traces in order to represent complete a program behavior. Although it is impractical to completely observe a complex program behavior, yet several software programs have been introduced to extend code coverage like Monkey Runner [30]. However, it is still argued [84,85] to effectively provide full behavior coverage with existing options.

## Conclusion and Future Work

Smartphones have become an attractive substitute for desktop computers because of the rapid development in compute intensive mobile phone technologies. The wide-scale deployment of Internet technologies (4G) enable smartphone users to be always connected to the network. Ultimately, these trends have opened the door for cybercriminals to expand their malevolent motivations towards recent evolving platform. New vulnerabilities have evolved with the extension of smartphone usage to general-purpose computing and production environments. The mobile botnet phenomenon is inherited from previous generation of PC-based botnets aiming to gain illegitimate access to mobile devices to carryout various malicious activities. We propose SMARTbot, a novel framework to analyze and detect potential Android-based mobile botnet applications through dynamic analysis augmented by machine learning techniques. The framework is decomposed into three components; dynamic analysis component, feature mining component and learning component. During dynamic analysis, applications are required to be executed in a secure sandbox and the results are collected for further classification. In the feature mining component the feature vector is extracted from the generated profiles of all applications and stored in a repository for learning. Finally, in the learning component the sample of a known botnet dataset are trained with the help of ANN model. In addition to that, class labeling for the large scale Drebin dataset is performed using a backpropagation model. Various machine learning classifiers are applied to determine the most suitable classification algorithm to draw a clear line between botnet and other types of malicious applications.

As a result of this study, we conclude that: (a) mobile applications having C&C features show certain regular communication patterns with respect to families they belong, i.e bots of a certain family have common properties when coordinating with their C&C server. In addition to that, continuous connections to the C&C servers often follow similar timing and behavioral patterns. (b) Static analysis alone is not sufficient to detect these malicious binaries because of code obfuscation techniques imposed by cybercriminals. (c) Dynamic analysis augmented with machine learning is an obvious option to identify botnet behavior in Android applications even with diverse feature vector space. (d) To date, mobile botnet dataset remains unavailable; therefore this research could provide the foundation for future studies in the domain of botnet anomaly detection in mobile environment.

In our future work, we plan to devise a hybrid on-device analysis system for the detection of bot behavior using machine learning classifiers. For this purpose, we will attempt to design and implement our own sandbox with rich UI capabilities providing deep code coverage which can ultimately avoid all the deficiencies inherited from traditional dynamic analysis sandboxes.

## References

[1] Strazzere T (2014) The new NotCompatible: Sophisticated and evasive threat harbors the potential to compromise enterprise networks.

[2] Schwartz MJ (2012) Zeus Botnet.

[3] Mahaffey K (2011) Security Alert: DroidDream Malware Found in Official Android Market.

[4] F-Secure (2009) Threat Description Worm: iPhoneOS/Ikee.B.

[5] Mullaney C (2012) Android.Bmaster: A Million-Dollar Mobile Botnet.

[6] Jiang X (2012) Security Alert: New TigerBot Malware Found in Alternative Android Markets.

[7] Unuchek R (2013) Obad.a Trojan Now Being Distributed via Mobile Botnets.

[8] Danchev D (2013) How cybercriminals create and operate Android-based botnets.

[9] Kelly G (2014) Report: 97% Of Mobile Malware Is On Android. This Is The Easy Way You Stay Safe.

[10] Perdisci R, Lanzi A, Lee W (2008) Classification of packed executables for accurate computer virus detection. Pattern Recognition Letters 29: 1941–1946.

[11] Wicherski G. pehash: A novel approach to fast malware clustering; 2009.

[12] Karim ME, Walenstein A, Lakhotia A, Parida L (2005) Malware phylogeny generation using permutations of code. Journal in Computer Virology 1: 13–23.

[13] Kolter JZ, Maloof MA (2006) Learning to detect and classify malicious executables in the wild. The Journal of Machine Learning Research 7: 2721–2744.

[14] Dullien T, Rolles R (2005) Graph-based comparison of executable objects (english version). SSTIC 5: 1–3.

[15] Flake H. Structural comparison of executable objects; 2004. pp. pages 161–173.

[16] Bayer U, Comparetti PM, Hlauschek C, Kruegel C, Kirda E. Scalable, Behavior-Based Malware Clustering; 2009. Citeseer. pp. 8–11.

[17] Bailey M, Oberheide J, Andersen J, Mao ZM, Jahanian F, Nazario J. Automated classification and analysis of internet malware; 2007 Springer pp. 178–197.

[18] Rieck K, Trinius P, Willems C, Holz T (2009) Automatic analysis of malware behavior using machine learning: TU, Professoren der Fak. IV.

[19] Ahmad Karim RS, Syed Adeel Ali Shah (2015) DeDroid: A Mobile Botnet Detection Approach Based on Static Analysis. The 7th International Symposium on UbiCom Frontiers—Innovative Research, Systems and Technologies. Beijing, China: IEEE.

[20] Technology VUo (2012) Andrubis-analysis of android apks.

[21] Arp D, Spreitzenbarth M, Hubner M, Gascon H, Rieck K. Drebin: Efficient and explainable detection of android malware in your pocket; 2014.

[22] Karim A (2016) SmartBot_Dataset.

[23] Sookhak M, Gani A, Talebian H, Akhunzada A, Khan SU, Buyya R, et al (2015) Remote Data Auditing in Cloud Computing Environments: A Survey, Taxonomy, and Open Issues. ACM Computing Surveys (CSUR) 47: 65.

[24] Sookhak M, Gani A, Khan MK, Buyya R (2015) Dynamic remote data auditing for securing big data storage in cloud computing. Information Sciences.

[25] Enck W, Gilbert P, Han S, Tendulkar V, Chun B-G, Cox LP, et al (2014) TaintDroid: an information-flow tracking system for realtime privacy monitoring on smartphones. ACM Transactions on Computer Systems (TOCS) 32: 5.

[26] Zheng C, Zhu S, Dai S, Gu G, Gong X, Han X, et al Smartdroid: an automatic system for revealing ui-based trigger conditions in android applications; 2012 ACM pp. 93–104.

[27] Wu D-J, Mao C-H, Wei T-E, Lee H-M, Wu K-P. Droidmat: Android malware detection through manifest and api calls tracing; 2012 IEEE pp. 62–69.

[28] Zhou Y, Wang Z, Zhou W, Jiang X. Hey, You, Get Off of My Market: Detecting Malicious Apps in Official and Alternative Android Markets; 2012.

[29] Zhang Y, Yang M, Xu B, Yang Z, Gu G, Ning P, et al Vetting undesirable behaviors in android apps with permission use analysis; 2013 ACM pp. 611–622.

[30] Developers A (2012) Monkeyrunner.

[31] Desnos A, Lantz P (2011) Droidbox: An android application sandbox for dynamic analysis.

[32] Sahs J, Khan L. A machine learning approach to android malware detection; 2012 IEEE pp. 141–147.

[33] Aafer Y, Du W, Yin H (2013) DroidAPIMiner: Mining API-level features for robust malware detection in android Security and Privacy in Communication Networks: Springer pp. 86–103.

[34] Chakradeo S, Reaves B, Traynor P, Enck W. Mast: triage for market-scale mobile malware analysis; 2013 ACM pp. 13–24.

[35] Wang X, Yang Y, Zeng Y (2015) Accurate mobile malware detection and classification in the cloud. SpringerPlus 4: 1–23. 26543718 10.1186/s40064-015-1356-1 PMC4628031

[36] Lindorfer M, Neugschwandtner M, Platzer C (2014) MARVIN: Efficient and Comprehensive Mobile App Classification Through Static and Dynamic Analysis.

[37] Sookhak M, Talebian H, Ahmed E, Gani A, Khan MK (2014) A review on remote data auditing in single cloud server: Taxonomy and open issues. Journal of Network and Computer Applications 43: 121–141.

[38] Sookhak M, Akhundzada A, Sookhak A, Eslaminejad M, Gani A, Khan MK, et al (2015) Geographic Wormhole Detection in Wireless Sensor Networks. PloS one 10. 10.1371/journal.pone.0115324 PMC4300191 25602616

[39] Yan L-K, Yin H. DroidScope: Seamlessly Reconstructing the OS and Dalvik Semantic Views for Dynamic Android Malware Analysis; 2012. pp. 569–584.

[40] Petsas T, Voyatzis G, Athanasopoulos E, Polychronakis M, Ioannidis S. Rage against the virtual machine: hindering dynamic analysis of android malware; 2014 ACM pp. 5.

[41] NVISO (2014) APKScan.

[42] Symantec (2012) Android.Fakenotify.

[43] Leyden J (2014) Secluded HijackRAT: Monster mobile malware multitool from HELL: Web Report.

[44] Symantec (2014) Android.Hippo.B.

[45] F-Secure (2014) Threat Description: TROJAN: ANDROID/OPFAKE.

[46] wyatt t (2011) Security Alert: Geinimi, Sophisticated New Android Trojan Found in Wild.

[47] Svajcer V (2011) Plankton malware drifts into Android Market.

[48] Lookout (2012) Security Alert: SpamSoldier.

[49] Lookout (2011) Security Alert: New Malware Found in Alternative Android Markets: DroidKungFu.

[50] Dietrich CJ, Rossow C, Freiling FC, Bos H, van Steen M, Pohlmann N. On Botnets that use DNS for Command and Control; 2011 IEEE pp. 9–16.

[51] Krmicek V (2011) Inspecting DNS Flow Traffic for Purposes of Botnet Detection. GEANT3 JRA2 T4 Internal Deliverable: 1–9.

[52] Kang BBH (2011) DNS-Based Botnet Detection Encyclopedia of Cryptography and Security: Springer pp. 362–363.

[53] Strazzere T, Wyatt T (2011) Geinimi trojan technical teardown. Lookout Mobile Security.

[54] Blagov M (2015) HTTP Flood: DDoS Attack Glossary.

[55] AB SC (2015) Trojan list sorted on trojan port.

[56] SpeedGuide (2015) Ports Database.

[57] Svajcer V (2014) Sophos Mobile Security Threat Report. Launched at Mobile World Congress.

[58] Python (2015) The ElementTree XML API.

[59] Goyvaerts J (2015) Regular Expressions Tutorial: Learn How to Use and Get The Most out of Regular Expressions.

[60] Muttik I. Malware mining, Virus Bulletin International Conference, VB2011; 2011; Barcelona, Spain.

[61] papagelis AJ (2013) Multi-Layer Perceptron.

[62] Bhargava N, Sharma G, Bhargava R, Mathuria M (2013) Decision tree analysis on j48 algorithm for data mining. Proceedings of International Journal of Advanced Research in Computer Science and Software Engineering 3.

[63] Quinlan JR. Bagging, boosting, and C4. 5; 1996. pp. 725–730.

[64] Landwehr N, Hall M, Frank E (2005) Logistic model trees. Machine Learning 59: 161–205.

[65] Pawlak Z (2002) Rough sets, decision algorithms and Bayes' theorem. European Journal of Operational Research 136: 181–189.

[66] Frank E, Hall M, Holmes G, Kirkby R, Pfahringer B, Witten IH, et al (2010) Weka-a machine learning workbench for data mining Data Mining and Knowledge Discovery Handbook: Springer pp. 1269–1277.

[67] Hall M, Frank E, Holmes G, Pfahringer B, Reutemann P, Witten IH (2009) The WEKA data mining software: an update. ACM SIGKDD explorations newsletter 11: 10–18.

[68] Refaeilzadeh P, Tang L, Liu H (2009) Cross-validation. Encyclopedia of database systems: Springer pp. 532–538.

[69] Melville P, Saar-Tsechansky M, Provost F, Mooney R. Active feature-value acquisition for classifier induction; 2004 IEEE pp. 483–486.

[70] McLachlan G, Do K-A, Ambroise C (2005) Analyzing microarray gene expression data: John Wiley & Sons.

[71] Weichselbaum L, Neugschwandtner M, Lindorfer M, Fratantonio Y, van der Veen V, Platzer C (2014) Andrubis: Android malware under the magnifying glass. Vienna University of Technology, Tech Rep TRISECLAB-0414-001.

[72] Wang X, Yu H (2005) How to break MD5 and other hash functions Advances in Cryptology–EUROCRYPT 2005: Springer pp. 19–35.

[73] Syverson P. A taxonomy of replay attacks [cryptographic protocols]; 1994 IEEE pp. 187–191.

[74] StackExchange (2014) How weak is MD5 as a password hashing function?.

[75] Ingram M (2015) Mobile botnets are costing advertisers $1 billion in ad fraud, study shows.

[76] Spreitzenbarth M, Freiling F, Echtler F, Schreck T, Hoffmann J. Mobile-sandbox: Having a deeper look into android applications; 2013 ACM pp. 1808–1815.

[77] Yerima SY, Sezer S, McWilliams G (2014) Analysis of Bayesian classification-based approaches for Android malware detection. IET Information Security 8: 25–36.

[78] Chuang H-Y, Wang S-D. Machine Learning Based Hybrid Behavior Models for Android Malware Analysis; 2015 IEEE pp. 201–206.

[79] Peiravian N, Zhu X. Machine learning for android malware detection using permission and api calls; 2013 IEEE pp. 300–305.

[80] Parkour M (2011) Contagio malware dump.

[81] Networks B (2015) Spam Data.

[82] Vidas T, Christin N. Evading android runtime analysis via sandbox detection; 2014 ACM pp. 447–458.

[83] Unuchek R (2013) Obad.a Trojan Now Being Distributed via Mobile Botnets. Available: http://securelist.com/blog/mobile/57453/obad-a-trojan-now-being-distributed-via-mobile-botnets/.

[84] Hutchins M, Foster H, Goradia T, Ostrand T. Experiments of the effectiveness of dataflow-and controlflow-based test adequacy criteria; 1994 IEEE Computer Society Press pp. 191–200.

[85] Inozemtseva L, Holmes R. Coverage is not strongly correlated with test suite effectiveness; 2014 ACM pp. 435–445.

